# Body composition and short-term mortality in patients critically ill with acute-on-chronic liver failure

**DOI:** 10.1016/j.jhepr.2023.100758

**Published:** 2023-04-07

**Authors:** Thomas Mangana del Rio, Sophie-Caroline Sacleux, Julien Vionnet, Philippe Ichaï, Alban Denys, Antoine Schneider, Audrey Coilly, Montserrat Fraga, Alexandre Wetzel, Joachim Koerfer, Jean-Daniel Chiche, Faouzi Saliba, Darius Moradpour, Fabio Becce, Florent Artru

**Affiliations:** 1 Division of Gastroenterology and Hepatology, Lausanne University Hospital and University of Lausanne, Lausanne, Switzerland; 2 Liver Intensive Care Unit, AP-HP Paul Brousse Hospital, University Paris SACLAY, INSERM Unit N°1193, Villejuif, France; 3 Transplantation Centre, Lausanne University Hospital and University of Lausanne, Lausanne, Switzerland; 4 Department of Diagnostic and Interventional Radiology, Lausanne University Hospital and University of Lausanne, Lausanne, Switzerland; 5 Division of Intensive Care Medicine, Lausanne University Hospital and University of Lausanne, Lausanne, Switzerland; 6 Data Science, Lausanne University Hospital, Lausanne, Switzerland; 7 Institute of Liver Studies, King’s College Hospital, London, UK

**Keywords:** Acute-on-chronic liver failure, Body composition, Cirrhosis, Intensive care medicine, Sarcopenia, Computed tomography, Skeletal muscle index, Subcutaneous adipose tissue radiation attenuation, Visceral-on-subcutaneous adipose tissue ratio

## Abstract

**Background & Aims:**

Body composition is sex dependent and associated with an increased mortality risk in patients with cirrhosis. We evaluated whether it was also associated with short-term mortality in patients critically ill with acute-on-chronic liver failure (ACLF).

**Patients and methods:**

We retrospectively included all patients with cirrhosis and ACLF hospitalised in the intensive care unit (ICU) of Lausanne University Hospital between 2010 and 2019 for whom an abdominal computed tomography (CT) scan performed ±7 days from admission was available. Patients from the ICU of Paul Brousse University Hospital admitted between 2017 and 2020 served as an external cohort. All body composition parameters at the third lumbar vertebral level (L3) were quantified using a deep learning-based method.

**Results:**

In total, 192 patients from Lausanne were included. Median age was 62 years and 28-day survival rate was 58.2%. In males, variables independently associated with 28-day mortality on days 1 and 3 were Chronic Liver Failure Consortium (CLIF-C) ACLF-lactate and sarcopenia. In females, CLIF-C ACLF-lactate on days 1 and 3 was the only predictor of 28-day survival. We derived two scores combining sarcopenia and the CLIF-C ACLF-lactate score on days 1 and 3, with area under the receiver operating characteristic outperforming the CLIF-C ACLF-lactate score alone in male but not in female patients. Comparable results were found in the external cohort of 58 patients and supported the sex specificity of the performance of the model. Patients with sarcopenia had increased risks of invasive fungal infection and renal replacement therapy.

**Conclusion:**

Sarcopenia was associated with 28-day mortality in male but not in female patients critically ill with ACLF. Although screening for sarcopenia could impact the management of male patients, further studies are needed in female cohorts to investigate whether other body composition parameters are associated with outcomes.

**Impact and implications:**

Body composition, easily assessed by CT, is altered in patients with cirrhosis and associated with outcome; it has never been investigated in patients critically ill with ACLF. The results of the present study, underlining the benefit of sarcopenia evaluation to improve prognosis prediction in males critically ill with ACLF, are of importance for physicians managing such patients to optimise the decision-making process toward continued treatment, liver transplantation, or limitation of care. In a wider sense, besides the number and course of organ failures, the results recall the weight of the general condition of males with ACLF at admission to ICU. In females critically ill with ACLF, in analyses limited by the sample size, none of the body composition parameters was associated with short-term mortality independently of organ failures; this suggests that the number and course of organ failures are the main determinant of mortality in these patients.

## Highlights

• Sarcopenia is independently associated with 28-day mortality in male but not in female patients critically ill with cirrhosis and ACLF.
• Incorporation of sarcopenia into the CLIF-C ACLF-lactate score improves the prediction of prognosis in male patients.
• Patients with sarcopenia have an increased risk of severe ACLF course and of fungal infection, as well as an increased risk of requiring renal replacement therapy.

## Graphical abstract

**Figure:**
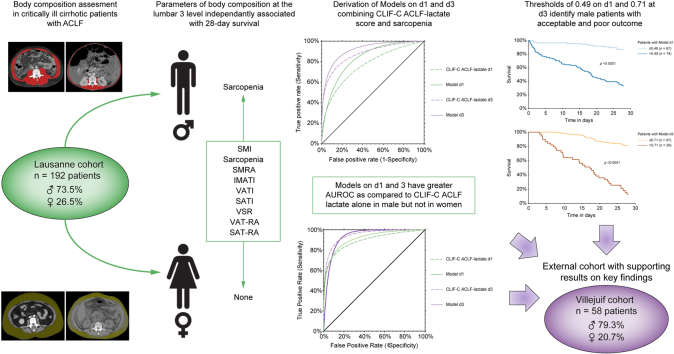

## Introduction

Acute-on-chronic liver failure (ACLF) affects up to 30–40% of patients hospitalised with cirrhosis.[1], [2], [3] This condition is associated with the development of organ failure (OF) and a high short-term mortality.[1], [2], [3] Patients with cirrhosis developing ACLF often require organ support in the intensive care unit (ICU). Over the past 10 years, it has been established that OF-based scores strongly predict the short-term mortality risk in this population.^2^^,^[4], [5], [6] Accordingly, scoring systems dedicated to patients with ACLF, namely the Chronic Liver Failure Consortium (CLIF-C) OF score and CLIF-C ACLF scores, have been developed and externally validated.^5^^,^^7^^,^^8^ Importantly, it was recently suggested that lactate is an independent prognostic marker in this population and that the CLIF-C ACLF-lactate score, which includes the serum lactate level at admission, outperforms the CLIF-C ACLF score in the prediction of short-term mortality.^7^^,^^9^ Finally, the predictive accuracy of these scoring systems is even greater after 3–7 days of medical management.^2^^,^^8^ Hence, it is essential to assess any additional potential factor related to short-term mortality not only upon admission to the ICU but also throughout the ICU stay for comprehensive evaluation.

Sarcopenia, a condition defined by the loss of skeletal muscle mass and function, is highly prevalent in patients with cirrhosis and affects up to 90% of patients awaiting liver transplantation (LT).^10^ Portosystemic shunting and subsequent hyperammonaemia leading to the synthesis of myostatin, a potent negative regulator of muscle growth, are among the main mechanisms involved in sarcopenia development.^11^ In addition, hormonal changes, such as low testosterone and growth hormone levels, also act as myostatin promoters. Moreover, chronic inflammation induced by hepatocellular necrosis and related damage-associated molecular pattern and pathogen-associated molecular pattern release participate in the proteolysis observed in these patients.[11], [12], [13] Finally, low dietary intake and depleted glycogen stores contribute to muscle catabolism.[12], [13], [14], [15] Sarcopenia has been associated with worse outcomes in patients with cirrhosis before and after LT.[16], [17], [18] It has also been associated with poorer outcomes in patients critically ill without cirrhosis[19], [20], [21], [22], [23] and is likely associated with prolonged intubation and failure of weaning from mechanical ventilation.^24^^,^^25^ Although possibly prevalent in patients hospitalised in the ICU with ACLF, the impact of sarcopenia on the outcomes of this population has not been examined so far.

Considering the ease of assessing sarcopenia by computed tomography (CT) and its potential impact on the outcome and management of these patients, we investigated the prevalence of sarcopenia and its association with short-term mortality.^26^ Besides sarcopenia, body composition itself is altered in cirrhosis and its components can be adequately differentiated on CT scans. Among them, the ratio of visceral and subcutaneous adipose tissue and radiodensity have been recently associated with outcomes before and after LT.[27], [28], [29] The analyses of the adipose tissue density expressed in Hounsfield units (HUs) can provide important information about the quality of the tissue and indirectly offer an insight into the pathophysiology. In fact, adipose tissue is central in hypercatabolic conditions, serving as a substrate and modulating energy metabolism. Several potential factors, such as blood flow, adipocyte size, lipid content, and fluid-to-triglyceride ratio, might impact radiodensity (also referred to as radiation attenuation) measured by CT in HU.^27^ Importantly, body composition is affected by sex, with an increased adipose tissue mass in women and an increased muscle mass in men. Such observations have also been reported in the setting of cirrhosis.^27^^,^^29^ Thus, any new approach investigating body composition parameters should include sex-based analyses. Therefore, taking into consideration patients’ sex, we aimed to investigate whether body composition parameters were associated with mortality and outcome in patients with cirrhosis critically ill with ACLF.

## Patients and methods

### Patients

**Fig. 1:**
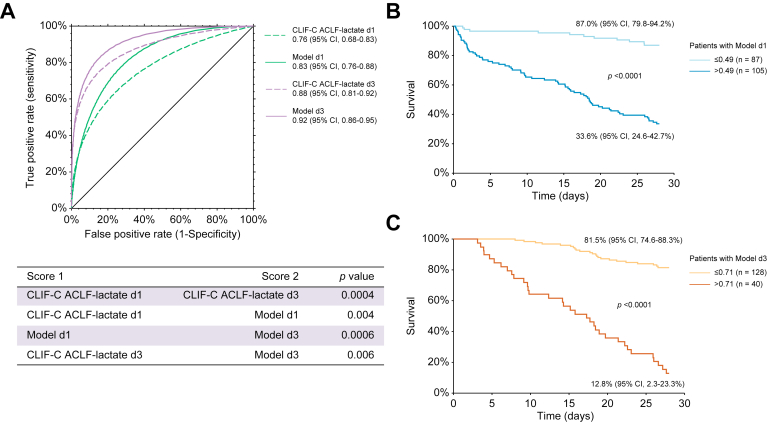
Performance of the available and newly developed models (Model d1 and d3) in the overall Lausanne cohort. (A) Receiver operating characteristic curves for survival at 28 days in the overall cohort as determined by the Chronic Liver Failure Consortium (CLIF-C) acute-on-chronic liver failure (ACLF)-lactate-sarcopenia score on Day 1 (Model d1, 0.83 [95% CI 0.76–0.88]) and Day 3 (Model d3, 0.92 [95% CI 0.86–0.95]) *vs.* the CLIF-C ACLF-lactate score at Day 1 (0.76 [95% CI 0.68–0.83], *p* = 0.004) and Day 3 (0.88 [95% CI 0.81–0.92], *p* = 0.006). (B) The 28-day Kaplan–Meier survival analysis of the overall cohort according to the CLIF-C ACLF-lactate-sarcopenia score at Day 1 (Model d1, cut-off ≤0.49). (C) 28-day Kaplan–Meier survival analysis of the overall cohort according to the CLIF-C ACLF-lactate-sarcopenia score at Day 3 (Model d3, cut-off ≤0.71). The 28-day survival was estimated using the Kaplan–Meier method and compared with the log-rank test. Survival was expressed as a percentage with 95% CI. The differences in terms of diagnostic accuracy between the models and the CLIF-C ACLF-lactate score on Days 1 and 3 were assessed by comparison of area under the receiver operating characteristic curves using the z test described by Zhou *et al.*^39^

Two cohorts of patients were included. In a first retrospective exploratory cohort, we included all patients with liver cirrhosis and ACLF admitted to the ICU of Lausanne University Hospital (Lausanne, Switzerland) between 1 January 2010, and 31 December 2019. In this cohort, patients were identified primarily through ICM-10 codes for liver disease (see supplementary data for the complete list) and reviewed to confirm the diagnosis of cirrhosis, which was based on clinical, laboratory, imaging, and histopathological features. In a second prospective external cohort, the primary aim of which was investigation of the selection process toward LT, all patients with liver cirrhosis and ACLF admitted to the liver ICU of Paul Brousse University Hospital (Villejuif, France) between July 2017 and March 2020 were included. In both cohorts the ACLF grade according to the European Association for the Study of the Liver classification was calculated for each patient.^3^ All patients with cirrhosis and ACLF grade 1, 2, or 3 were included according to availability at admission ±7 days of a CT, allowing evaluation of the body composition parameters in the exploratory cohort from Lausanne (Fig. 1). In the external cohort from Villejuif, all patients with cirrhosis and ACLF grade 1, 2, or 3 were included according to the availability of a CT from -5 to +2 days from admission, allowing evaluation of their body composition parameters. This timeframe was chosen because 90% of CT scans were performed within this timeframe in the exploratory cohort from Lausanne (see below). The study was approved by the two local ethical committees (Ethical Committee of the Canton de Vaud [CER-VD, protocol number 2020-02691] and Centre de Protection des Personnes – IDF – Bicêtre) in accordance with the International Guideline for Ethical Review of Epidemiological Studies and principles of the Declaration of Helsinki.

### Clinical and laboratory data

The following clinical and laboratory data were retrospectively collected from medical records:

At the time of admission to the ICU*:* age, height, weight, body mass index (BMI), ethnicity, cause of cirrhosis, main comorbidities (cancer, diabetes, arterial hypertension, chronic cardiac, or respiratory or kidney disease), frailty (clinical frailty scale), reason for ICU admission, medical treatment on admission, organ support [mechanical ventilation, vasopressors, renal replacement therapy (RRT)], presence of ascites, hepatic encephalopathy, leukocytes count, international normalised ratio (INR), bilirubin, transaminases level, albumin, creatinine, sodium, arterial lactate level, ammonia, C-reactive protein (CRP) as well as severity scores for cirrhosis (Child-Pugh and Model for End-Stage Liver Disease [MELD]) and ACLF (ACLF grade based on CLIF-OF, CLIF-C ACLF, or CLIF-C ACLF-lactate).

During the course of the ICU stay: organ supports, presence of ascites, hepatic encephalopathy, leukocytes count, INR, bilirubin, transaminase level, albumin, creatinine, sodium, arterial lactate level, ammonia, CRP, severity scores for cirrhosis and ACLF, treatments (including nutritional support and mean calorie intake), occurrence and site of bacterial (see definitions in the supplementary data) and/or probable/proven fungal invasive infection,^30^ death and cause of death, LT and date of LT, date of discharge from the ICU, and date of last follow-up.

### Assessment of body composition

The following body composition parameters were assessed from a single axial CT image of the abdomen at the third lumbar vertebral level (L3) using a semiautomated method: skeletal muscle area (SMA, in cm^2^), skeletal muscle radiation attenuation (SMRA, in HU), intermuscular adipose tissue area (IMAT, in cm^2^), subcutaneous adipose tissue area (SAT, in cm^2^), visceral adipose tissue area (VAT, in cm^2^), subcutaneous adipose tissue area radiation attenuation (SAT-RA, in HU), and visceral adipose tissue area radiation attenuation (VAT-RA, in HU). All areas were normalised by patient height (m^2^), resulting in the following indices in cm^2^/m^2^: L3SMI, IMAT, SATI, and VATI. Visceral-on-subcutaneous adipose tissue area ratio (VSR) was calculated by dividing VATI by SATI.

The deep learning-based method applied in this study followed a traditional U-Net architecture, which was modified by adding a second, smaller U-Net to improve its accuracy.^31^ This method has been tested and validated in large CT data sets and has proven to be accurate and reliable.[32], [33], [34] The specific tissue demarcation used standard HU thresholds of -29 to +150 HU for skeletal muscle, -150 to -50 HU for VAT, and -190 to -30 HU for SAT. All automated segmentations were secondarily reviewed and adjusted where necessary by an expert musculoskeletal radiologist (F.B.) blinded to the patient's outcome, using a custom free-hand graphical user interface. Sarcopenia was defined as L3SMI ≤50 cm^2^/m^2^ in men and ≤39 cm^2^/m^2^ in women.^35^^,^^36^

### Statistical analyses

Quantitative variables were expressed as median (IQR). Categorical variables were expressed as frequencies and percentages. The primary endpoint was 28-day survival and was estimated using the Kaplan–Meier method and compared with the log-rank test. Survival was expressed as a percentage with 95% CI. Patients who underwent LT were censored alive at the time of LT. Uni- and multivariable logistic regression was performed at two time points (Day 1 and Day 3) to identify variables associated with the primary endpoint. Variables with *p* ≤0.1 were included in the multivariable analysis. The significance level was set at 0.05 with a 2-sided test. In these analyses, all variables with *p* ≤0.1 and included in the severity scores were not included in the multivariable analysis to avoid collinearity. Similarly, the severity score with the best odds ratio (OR) was included in the multivariable analysis.

To better stratify the risk of mortality in patients critically ill with cirrhosis, we developed two predictive models that included the factors independently associated with 28-day mortality at Days 1 and 3 according to the following statistical methodology: to obtain a probability score ranging from 0 to 1, the R function obtained by the forward logistic regression function combining the most discriminatory independent factors was inserted into formula 1/(1 + Exp[−R]), as previously described.^37^^,^^38^ The prediction of the model was expressed in both cohorts from Lausanne and Villejuif using the area under the receiver operating characteristic curve (AUROC) with the percentage of patients correctly classified. Calibration of the scores was assessed in both cohorts from Lausanne and Villejuif with the Hosmer–Lemeshow test to confirm similar observed and predicted 28-day mortality. The differences in terms of diagnostic accuracy between the models and the CLIF-C ACLF-lactate score at Days 1 and 3 were assessed in both cohorts from Lausanne and Villejuif by comparison of AUROCs using the z test described by Zhou *et al.*^39^ Comparisons between male and female patients as well as patients with and without sarcopenia were performed using the Student’s *t* test or Mann-Whitney *U* test for quantitative variables or Chi-square and Fisher exact tests for categorical variables, as appropriate. All statistical analyses were performed using NCSS 2022 software (NCSS 2022 Statistical Software (2022). NCSS, LLC. Kaysville, Utah, USA, ncss.com/software/ncss.) and MedCalc 20-115 (MedCalc Software Ltd).

## Results

### Main characteristics of the Lausanne exploratory cohort

A total of 192 patients admitted to the ICU of the Lausanne University Hospital fulfilled the inclusion criteria and were included in the analyses of the exploratory cohort (Fig. S1). The median age was 62.0 years (IQR 53.2–70.0 years), 141 patients (73.5%) were males, and 161 patients (83.8%) were White. The main reasons for ICU admission were sepsis (n = 79; 41.1%) and gastrointestinal bleeding (n = 63; 32.8%). ACLF grades at admission were as follows: 26 patients (13.6%) had ACLF grade 1, 59 patients (30.7%) ACLF grade 2, and 107 patients (55.7%) ACLF grade 3. The 28-day survival rate was 58.2% (95% CI 51.2–65.2). Fourteen patients (7.3%) underwent LT during follow-up and only one (0.5%) was transplanted at 28 days. The 28-day survival rate was 93.2% (88.8–100.0), 72.0% (59.5–80.4), and 40.6% (31.2–49.9) in patients with ACLF grade 1, 2, and 3, respectively (*p* <0.0001). Comparisons with main characteristics on Day 1 of patients who were not included in the final analyses (CT not performed or absence of CT slice allowing for body composition evaluation, n = 240) are provided in Table S1. No differences were found between the two populations except for an increase in leukocyte counts in patients included in the final analyses 15.0 G/L (10.1–20.8) *vs.* 13.6 G/L (9.7–19.3), *p* = 0.04 and CRP level (55.0 mg/L [20.0–112.5] *vs.* 39.0 mg/L [13.0-92.0], *p* = 0.02).

The median time interval between ICU admission and CT allowing body composition assessment was 0 days (IQR -2 to +1 days). Of note, 90% of CT scans were performed between 5 days before admission and 2 days after admission. According to L3SMI sex-specific cut-offs, 121 patients (63.0%) were sarcopenic. The body composition parameters differed between male and female patients in terms of L3SMI (47.1 cm^2^/m^2^ [39.5–52.9] *vs.* 38.7 cm^2^/m^2^ [32.2–41.0], *p* <0.0001), SMRA (38.0 HU [32.5–42.5] *vs.* 32.0 HU [28.2–36.0], *p* <0.0001), IMATI (5.9 cm^2^/m^2^ [3.6–9.2] *vs.* 8.75 cm^2^/m^2^ [5.1–12.0], *p* = 0.01) and SATI (44.5 cm^2^/m^2^ [27.8–66.9] *vs.* 53.1 cm^2^/m^2^ [25.3–95.5], *p* = 0.03) (Table 1). None of the other clinical and biological characteristics differed between male and female patients.

**Table 1:** **Characteristics of patients from the Lausanne cohort at Day 1 and Day 3**∗.

Characteristics	Overall population (n = 192)	Male patients (n = 141)	Female patients (n = 51)	*p* value
Age (years)	62.0 (53.2–70.0)	62.0 (53.0–69.0)	67.0 (56.0–73.0)	0.11
Sex (male)	141 (73.5)	—	—	—
Body mass index (kg/m^2^)	25.8 (22.3–31.2)	26.6 (23.3–31.0)	24.0 (20.3–28.7)	0.08
Ethnicity				0.36
Caucasian	161 (83.8)	116 (82.2)	45 (88.2)	
Hispanic	16 (8.3)	13 (9.2)	4 (7.8)	
Other	15 (7.8)	12 (8.5)	2 (3.9)	
Clinical frailty score	4.0 (3.0–4.0)	4.0 (3.0–4.0)	4.0 (3.5–5.0)	0.21
Aetiology				
Alcohol	129 (67.2)	93 (66.0)	36 (70.6)	0.09
Viral	34 (17.7)	30 (21.3)	4 (7.8)	
Metabolic	18 (9.4)	12 (8.5)	6 (11.8)	
Other	11 (5.7)	6 (4.3)	5 (9.8)	
Reason for ICU admission				
Sepsis	79 (41.1)	60 (42.9)	19 (37.3)	0.08
Bleeding	63 (32.8)	49 (34.8)	14 (27.5)	
Other	50 (26.1)	32 (22.7)	18 (34.6)	

**Characteristics on Day 1**				
Sample size	n = 192	n = 141	n = 51	
Laboratory on Day 1				
Leukocytes (G/L)	15.0 (10.1–20.8)	15.3 (10.4–21.0)	14.1 (10.9–19.9)	0.91
International normalised ratio	1.5 (1.3–1.8)	1.4 (1.2–1.8)	1.6 (1.4–2 0.0)	0.08
Bilirubin (mg/dl)	5.3 (3.7–8.2)	5.1 (3.5–8.3)	5.6 (4.2–8.0)	0.99
Aspartate aminotransferase (IU)	93.0 (48.0–299.5)	83.0 (47.0–257.0)	143 (57.0–675.0)	0.91
Albumin (g/L)	27.0 (23.2–31.0)	28.0 (24.0–31.0)	26.0 (23.0–31.0)	0.81
Creatinine (mg/dl)	1.4 (1.0–2.1)	1.4 (1.0–2.1)	1.4 (0.7–2.2)	0.58
Sodium (mmol/L)	138.0 (134.0–141.0)	138.0 (134.0–142.0)	138.0 (134.0–141.0)	0.50
Lactate (mmol/L)	4.1 (2.4–7.4)	4.0 (2.2–6.6)	4.5 (2.7–8.2)	0.07
Ammonia (μmol/L)	71.0 (53.0–112.0)	72 (53–112)	68.5 (56.8–111.8)	0.82
C-reactive protein (mg/L)	55.0 (20.0–112.5)	54.0 (16.0–116.0)	61.0 (20.0–108.0)	0.93
Organ failure on Day 1				
Liver	52 (27)	38 (26.9)	14 (27.4)	0.78
Kidney	63 (32.8)	44 (31.2)	19 (37.3)	0.43
Brain	58 (30.2)	44 (31.2)	14 (27.5)	0.61
Coagulation	37 (19.3)	25 (17.7)	12 (23.5)	0.36
Circulation	168 (87.5)	126 (89.4)	42 (82.4)	0.19
Lung	94 (49.0)	69 (48.9)	25 (49.0)	0.99
Organ support on Day 1				
Renal replacement therapy	24 (12.5)	16 (11.3)	8 (15.7)	0.42 0.19 0.58
Vasopressors	168 (87.5)	126 (89.4)	42 (82.4)	
Mechanical ventilation	104 (54.2)	79 (56.0)	25 (49.0)	
ACLF grade on Day 1				
0	0 (0)	0 (0)	0 (0)	0.42
1	26 (13.6)	19 (13.5)	7 (17.7)	
2	59 (30.7)	45 (31.9)	14 (27.5)	
3	107 (55.7)	77 (58.8)	30 (58.8)	
Scores on Day 1				
MELD	21.9 (15.1–27.9)	21.5 (15.3–27.7)	23.5 (14.5–26.9)	0.64
CLIF-C ACLF	67.5 (51.8–72.7)	67.1 (61.7–72.7)	69.5 (62.0–72.8)	0.38
CLIF-C ACLF lactate	71.5 (64.1–80.2)	70.8 (62.9–79.4)	73.2 (67.7–83.0)	0.07

**Characteristics on Day 3**				
Sample size	n = 168	n = 126	n = 42	
Laboratory on Day 3				
Leukocytes (G/L)	12.6 (8.5–17.9)	11.0 (8.3–15.8)	13.5 (7.7–18.4)	0.90
International normalised ratio	1.4 (1.2–1.9)	1.4 (1.2–1.8)	1.4 (1.3–1.7)	0.51
Bilirubin (mg/dl)	6.0 (4.1–11.9)	6.0 (4.0–11.6)	6.0 (4.3–12.8)	0.99
Aspartate aminotransferase (IU)	93.0 (53.0–202.3)	82.0 (52.0–145.0)	91.0 (50.0–165.0)	0.54
Albumin (g/L)	27.0 (24.0–31.0)	27.0 (24.0–31.0)	28.0 (24.0–32.0)	0.27
Creatinine (mg/dl)	1.2 (0.8–1.9)	1.1 (0.8–1.7)	1.1 (0.6–1.8)	0.74
Sodium (mmol/L)	138.0 (135.0–142.0)	138.0 (135.0–142.0)	138.0 (135.0–142.0)	0.57
Lactate (mmol/L)	2.4 (1.7–3.7)	2.0 (1.6–3.1)	2.3 (1.8–3.3)	0.20
C-reactive protein (mg/L)	49.0 (24.0–113.0)	47.0 (24.0–112.0)	52.0 (32.0–93.0)	0.97
Organ failure on Day 3				
Liver	42 (5.0)	28 (22.2)	14 (33.3)	0.17
Kidney	36 (21.4)	27 (21.4)	9 (21.4)	0.99
Brain	42 (25.0)	32 (25.4)	10 (23.8)	0.78
Coagulation	27 (16.1)	21 (16.7)	6 (14.3)	0.63
Circulation	82 (48.8)	60 (47.6)	22 (52.3)	0.59
Lung	101 (59.5)	76 (60.3)	25 (59.5)	0.79
Organ support on Day 3				
Renal replacement therapy	21 (13.1)	17 (10.1)	4 (2.4)	0.50
Vasopressors	82 (48.8)	60 (47.6)	242 (52.3)	0.59
Mechanical ventilation	101 (60.1)	72 (57.1)	29 (69.1)	0.17
ACLF grade on Day 3				
0	26 (15.5)	20 (15.9)	6 (14.2)	0.95
1	48 (28.6)	35 (27.8)	13 (31.0)	
2	50 (29.8)	37 (29.3)	13 (31.0)	
3	44 (26.2)	34 (27.0)	10 (23.8)	
Scores on Day 3				
MELD	20.2 (12.8–26.9)	18.5 (13.5–26.8)	22.0 (11.2–27.6)	0.90
CLIF-C ACLF	63.8 (57.3–72.1)	63.4 (56.2–69.6)	62.0 (57.6–72.2)	0.72
CLIF-C ACLF-lactate	64.4 (56.2–73.3)	62.5 (55.1–69.2)	62.5 (55.3–72.5)	0.52

**Outcome**				
28-day survival, % (95% CI)	58.2 (51.2–65.2)	59.2 (50.6–67.7)	54.5 (42.1–67.0)	0.50

**Body composition parameters**				
L3SMI (cm^2^/m^2^)	43.2 (37.1–50.1)	47.1(39.5–52.9)	38.7 (32.2–41.0)	<0.0001
Sarcopenia according to L3SMI sex-specific cut-offs	121 (63.0)	84 (65.6)	37 (57.8)	0.30
SMRA (HU)	36.0 (31.0–41.0)	38.0 (32.5–42.5)	32.0 (28.2–36.0)	<0.0001
IMATI (cm^2^/m^2^)	6.3 (3.9–10.0)	5.9 (3.6–9.2)	8.75 (5.1–12.0)	0.01
VATI (cm^2^/m^2^)	44.3 (24.4–69.9)	47.9 (25.9–75.7)	38.4 (22.7–61.1)	0.11
SATI (cm^2^/m^2^)	48.6 (27.0–70.3)	44.5 (27.8–66.9)	53.1 (25.3–95.5)	0.03
VSR	0.9 (0.6–1.4)	1.0 (0.7–1.5)	0.6 (0.4–1.1)	0.07
VAT-RA (HU)	-81.2 (-88.5 to -75.8)	-81.3 (-88.7 to -75.8)	-80.1 (-86.1 to -75.8)	0.47
SAT-RA (HU)	-86.6 (-95.7 to -76.8)	-88.4 (-97.5 to -78.5)	-82.3 (-92.3 to -73.5)	0.09

ACLF, acute-on-chronic liver failure; CLIF-C, chronic liver failure consortium; HU, Hounsfield unit; ICU, intensive care unit; IMATI, intermuscular adipose tissue area index; MELD, Model for End-Stage Liver Disease; SATI, subcutaneous adipose tissue area index; SMRA, skeletal muscle radiation attenuation; SAT-RA, subcutaneous adipose tissue area radiation attenuation; VAT-RA, visceral adipose tissue area radiation attenuation; VATI, visceral adipose tissue area index; VSR, visceral-on-subcutaneous adipose tissue area ratio.

∗ Overall cohort, n = 192; males, n = 141; females, n = 64. Continuous and categorical variables expressed in median (IQR) and n (%), respectively. Comparisons were performed using the Student’s *t* test or Mann-Whitney *U* test for quantitative variables or Chi-square and Fisher exact tests for categorical variables as appropriate.

Comparisons with main characteristics at Day 1 of patients without ACLF admitted to the ICU who underwent a CT allowing for body composition assessment at admission ±7 days (n = 131) are provided in Table S2. Patients without ACLF were mainly admitted to the ICU in the context of gastrointestinal bleeding and postoperative surveillance. Compared with patients with ACLF, patients without ACLF were less sarcopenic (46.5% *vs.* 63.0%, *p* = 0.01) with higher L3SMI (46.8 cm^2^/m^2^ [36.3–57.8] *vs.* 43.2 cm^2^/m^2^ [37.1–50.1], *p* = 0.009). Moreover, these patients had increased VATI (49.4 cm^2^/m^2^ [22.6–87.1] *vs.* 44.3 cm^2^/m^2^ [24.4–69.9], *p* = 0.03), SATI (53.0 cm^2^/m^2^ [31.1–78.9] *vs.* 48.6 cm^2^/m^2^ [27.0–70.3], *p* = 0.01), and decreased VAT-RA (-84.5 HU [-92.8 to -78.8] *vs.* -81.2 HU [-88.5 to -75.8], *p* = 0.002) and SAT-RA -88.3 HU [-100.1 to -75.2] *vs.* -88.3 HU [-100.1 to -75.2], *p* = 0.02) compared with patients with ACLF.

### Factors associated with 28-day mortality in the Lausanne exploratory cohort

In a first step, uni- and multivariable analyses of the prognostic values for 28-day mortality of clinical and laboratory variables on Days 1 and 3 were performed for the entire cohort (Table 2, Table 3). On Days 1 and 3, the two variables independently associated with 28-day mortality were CLIF-C ACLF-lactate (OR 1.11, 95% CI 1.06–1.16, *p* <0.0001 and OR 1.17, 95% CI 1.10–1.25, *p* <0.0001, respectively) and sarcopenia according to L3SMI cut-offs (OR 2.76, 95% CI 1.07–7.11, *p* = 0.02 and OR 2.69, 95% CI 1.03–7.57, *p* = 0.04, respectively). In male patients, similar results were obtained on Day 1, whereas on Day 3, CLIF-C ACLF-lactate was the only variable associated with 28-day mortality (Tables S3 and S5). In female patients, in addition to CLIF-C ACLF-lactate, SAT-RA was the only parameter associated with 28-day mortality on Day 1 in univariable analysis but was not significant in the multivariable model. On Day 3 in female patients, CLIF-C ACLF-lactate was the only variable associated with 28-day mortality (Tables S4 and S6).

**Table 2:** **Univariable and multivariable logistic regression analyses of predictors of 28-day mortality in the overall Lausanne cohort (n** = **192) on day 1**.

Covariate	OR	95% CI	*p* value	OR	95% CI	*p* value
Characteristics						
Age (years)	1.01	0.98–1.03	0.52			
Sex (male)	0.46	0.27–1.04	0.07	0.68	0.27–1.68	0.52
Body mass index (kg/m^2^)	1.02	0.96–1.18	0.51			
Ethnicity						
Caucasian	—	—	—			
Hispanic	2.2	0.87–4.03	0.11			
Other	0.71	0.21–5.03	0.73			
Clinical frailty score	1.05	0.85–1.98	0.16			
Aetiology						
Alcohol	—	—	—	—	—	—
Viral	1.92	0.90–4.15	0.09	1.85	0.61–5.53	0.27
Metabolic	0.76	0.26–2.16	0.62	0.86	0.17–4.30	0.85
Other	0.87	0.24–3.13	0.83	0.58	0.11–3.05	0.52
Reason for ICU admission						
Sepsis	—	—	—	—	—	—
Bleeding	0.47	0.24–0.95	0.04	1.21	0.45–3.23	0.69
Other	0.70	0.34–1.45	0.34	1.23	0.39–3.85	0.71
Laboratory						
Leukocytes (G/L)	1.02	0.99–1.05	0.11			
International normalised ratio	2.42	1.43–4.07	0.0009			
Bilirubin (mg/dl)	1.10	1.04–1.17	0.001			
Aspartate aminotransferase (IU)	1.01	1.00–1.01	0.06			
Albumin (g/L)	0.97	0.92–1.01	0.18			
Creatinine (mg/dl)	1.36	1.03–1.80	0.03			
Sodium (mmol/L)	0.99	0.95–1.03	0.78			
Lactate (mmol/L)	1.15	1.08–1.23	<0.0001			
Ammonia (μmol/L)	1.00	0.99–1.01	0.28			
C-reactive protein (mg/L)	1.00	0.99–1.01	0.57			
Organ failure						
Liver	4.04	1.59–10.30	0.003			
Kidney	2.11	1.14–3.90	0.01			
Brain	1.04	0.88–1.50	0.17			
Coagulation	3.78	1.76–8.12	0.0006			
Circulation	4.13	1.35–12.60	0.01			
Lung	2.15	1.19–3.86	0.01			
Organ support						
RRT	2.64	1.15–6.38	0.01			
Vasopressors	4.13	1.35–12.60	0.01			
Mechanical ventilation	5.24	1.92–14.23	0.002			
Scores						
MELD	1.09	1.05–1.14	<0.0001			
ACLF grade	4.52	2.58–7.92	<0.0001			
CLIF-C ACLF	1.11	1.06–1.16	<0.0001			
CLIF-C ACLF-lactate	1.15	1.09–1.19	<0.0001	1.11	1.06–1.16	<0.0001
Body composition parameters						
L3SMI (cm^2^/m^2^)∗	0.96	0.93–0.99	0.04	0.97	0.93–1.00	0.06
Sarcopenia (L3SMI cut-offs)∗	3.20	1.68–6.09	0.0004	2.76	1.07–7.11	0.02
SMRA (HU)	0.97	0.94–1.01	0.28			
IMATI (cm^2^/m^2^)	0.98	0.94–1.03	0.57			
VATI (cm^2^/m^2^)	0.99	0.98–1.01	0.45			
SATI (cm^2^/m^2^)	1.00	0.99–1.01	0.52			
VSR	0.79	0.46–1.34	0.39			
VAT-RA (HU)†	1.04	1.01–1.08	0.02	1.04	0.99–1.19	0.08
SAT-RA (HU)†	1.03	1.01–1.06	0.03	1.03	0.98–1.08	0.08
SAT-RA (HU), according to (27)	1.74	0.83–3.62	0.15			

ACLF, acute-on-chronic liver failure; CLIF-C, chronic liver failure consortium; HU, Hounsfield unit; ICU, intensive care unit; IMATI, intermuscular adipose tissue area index; MELD, Model for End-Stage Liver Disease; SATI, subcutaneous adipose tissue area index; SMRA, skeletal muscle radiation attenuation; SAT-RA, subcutaneous adipose tissue area radiation attenuation; VAT-RA, visceral adipose tissue area radiation attenuation; VATI, visceral adipose tissue area index; VSR, visceral-on-subcutaneous adipose tissue area ratio.

∗ Not included in the same multivariable analysis to avoid collinearity;

† Not tested in the same multivariable analysis due to collinearity (Spearman rank correlation coefficient: 0.74; degrees of freedom 139, *p* <0.0001).

**Table 3:** **Univariable and multivariable logistic regression analyses of predictors of 28-day mortality in the overall Lausanne cohort (n** = **168) on day 3**.

Covariate	Univariable analysis			Multivariable analysis		
	OR	95% CI	*p* value	OR	95% CI	*p* value
Characteristics						
Age (years)	1.00	0.97–1.03	0.75			
Sex (male)	0.78	0.37–1.61	0.51			
Body mass index (kg/m^2^)	1.00	0.93–1.07	0.93			
Ethnicity						
Caucasian	—	—	—			
Hispanic	2.00	0.91–3.87	0.14			
Other	0.95	0.61–4.35	0.87			
Clinical frailty score	1.14	0.86–1.95	0.15			
Aetiology						
Alcohol	—	—	—	—	—	—
Viral	2.29	1.01–5.21	0.04	2.51	0.79–7.97	0.13
Metabolic	1.14	0.39–3.30	0.78	2.50	0.55–11.28	0.23
Other	0.76	0.14–3.98	0.75	0.16	0.01–2.65	0.20
Cause for ICU admission						
Sepsis	—	—	—	—	—	—
Bleeding	0.46	0.21–0.99	0.05	0.57	0.18–1.79	0.33
Other	0.61	0.27–1.39	0.24	0.53	0.16–1.70	0.26
Laboratory						
Leukocytes (G/L)	1.09	1.03–1.15	0.0008			
International normalised ratio	5.67	2.49–12.90	<0.0001			
Bilirubin (mg/dl)	1.07	1.02–1.13	0.004			
Aspartate aminotransferase (IU)	1.00	0.99–1.00	0.11			
Albumin (g/L)	1.06	0.97–1.14	0.15			
Creatinine (mg/dl)	1.38	1.04–1.82	0.02			
Sodium (mmol/L)	0.99	0.94–1.05	0.98			
Lactate (mmol/L)	1.50	1.20–1.88	0.0004			
Ammonia (μmol/L)	1.00	0.99–1.01	0.55			
C-reactive protein (mg/L)	1.00	0.99–1.01	0.90			
Organ failure						
Liver	3.57	1.60–7.93	0.002			
Kidney	2.38	1.12–5.06	0.02			
Brain	2.03	0.90–7.36	0.09			
Coagulation	8.16	3.17–20.95	<0.0001			
Circulation	3.42	1.74–6.74	0.0004			
Lung	2.32	1.15–5.68	0.009			
Organ support						
RRT	4.83	1.82–12.81	0.0009			
Vasopressors	3.42	1.74–6.74	0.0004			
Mechanical ventilation	3.20	1.55–6.60	0.0009			
Scores						
MELD	1.12	1.07–1.18	<0.0001			
ACLF grade	2.86	1.91–4.28	<0.0001			
CLIF-C ACLF	1.19	1.12–1.26	<0.0001			
CLIF-C ACLF lactate	1.20	1.13–1.27	<0.0001	1.17	1.10–1.25	<0.0001
Body composition parameters						
L3SMI (cm^2^/m^2^)∗	0.97	0.94–0.99	0.05	0.98	0.94–1.01	0.08
Sarcopenia (L3SMI cut-offs)∗	3.42	1.63–7.16	0.001	2.69	1.03–7.57	0.04
SMRA (HU)	0.99	0.95–1.04	0.88			
IMAT (cm^2^/m^2^)	0.96	0.90–1.02	0.23			
VAT (cm^2^/m^2^)	0.99	0.98–1.01	0.21			
SAT (cm^2^/m^2^)	1.00	0.98–1.01	0.97			
VSR	0.87	0.49–1.54	0.63			
VAT-RA (HU)†	1.06	1.01–1.11	0.02	1.04	0.97–1.12	0.20
SAT-RA (HU)†^,^‡	1.03	1.01–1.06	0.03	1.04	0.98–1.09	0.08
SAT-RA (HU)‡, according to^27^	2.14	0.97–4.70	0.06	3.00	0.90–10.12	0.09

ACLF, acute-on-chronic liver failure; CLIF-C, chronic liver failure consortium; HU, Hounsfield unit; ICU, intensive care unit; IMATI, intermuscular adipose tissue area index; MELD, Model for End-Stage Liver Disease; OR, odds ratio; SATI, subcutaneous adipose tissue area index; SMRA, skeletal muscle radiation attenuation; SAT-RA, subcutaneous adipose tissue area radiation attenuation; VAT-RA, visceral adipose tissue area radiation attenuation; VATI, visceral adipose tissue area index; VSR, visceral-on-subcutaneous adipose tissue area ratio.

∗ Not included in the same multivariable analysis to avoid collinearity;

† Not included in the same multivariable analysis to avoid collinearity (Spearman rank correlation coefficient: 0.75, degrees of freedom 123, *p* <0.0001);

‡ Not included in the same multivariable analysis to avoid collinearity.

### Model development in the overall Lausanne exploratory cohort

In the overall cohort, the final logistic regression functions combined the variables independently associated with 28-day mortality on day 1 (CLIF-C ACLF-lactate at day 1 and sarcopenia according to sex-specific L3SMI cut-offs) and day 3 (CLIF-C ACLF-lactate at day 3 and sarcopenia according to sex-specific L3SMI cut-offs).

The following formulas were obtained:

(1) R function of the Model d1: 7.51 – 0.09 × (CLIF-C ACLF-lactate at day 1) – 1.55 × (sarcopenia according to sex-specific L3SMI cut-offs [0 or 1]).

and

(2) R function of the Model d3: 10.57 – 0.15 × (CLIF-C ACLF-lactate at day 3) – 0.90 × (sarcopenia according to sex-specific L3SMI cut-offs [0 or 1]).

The output results for Model d1 and d3 ranged from 0 to 1. For these two models, the Hosmer–Lemeshow Chi-square statistic was 8.8 (8 degrees of freedom [df], *p* = 0.35) and 10.3 (8 df, *p* = 0.25), respectively, confirming similar observed and predicted 28-day mortality rate across 10 stratified groups. Overall, 74.5% and 83.0% of patients were correctly classified with Model d1 and Model d3. The AUROCs for 28-day survival probability (Fig. 1A) were 0.83 (95% CI 0.76–0.88) and 0.92 (95% CI 0.86–0.95), respectively. The AUROC of Model d1 was significantly higher than that of the CLIF-C ACLF-lactate score on Day 1 (0.76 [95% CI 0.68–0.83], *p* = 0.004). Similarly, the AUROC of the Model d3 was significantly higher than that of the CLIF-C ACLF-lactate score on day 3 (0.88 [95% CI 0.81–0.92], *p* = 0.006) (Fig. 1A). According to the Youden index, the best cut-offs for Model d1 and Model d3 were 0.49 and 0.71, respectively. With these cut-offs, the sensitivity was 88% and 71%, specificity 68% and 95%, positive predictive value 64% and 91%, negative predictive value 89% and 83%, and the percentage of patients correctly classified was 75% and 86%, respectively. According to these cut-offs, in the overall cohort, patients with a Model d1 ≤0.49 had a 28-day survival rate of 87.0% (95% CI 79.8–94.2%) *vs.* 33.6% (95% CI 24.6–42.7%) for those with a score >0.49 (*p* <0.0001) (Fig. 1B). In parallel, patients with a Model d3 ≤0.71 had a 28-day survival rate of 81.5% (95% CI 74.6–88.3%) *vs.* 12.8% (95% CI 2.3–23.3%) (*p* <0.0001) (Fig. 1C).

**Fig. 2:**
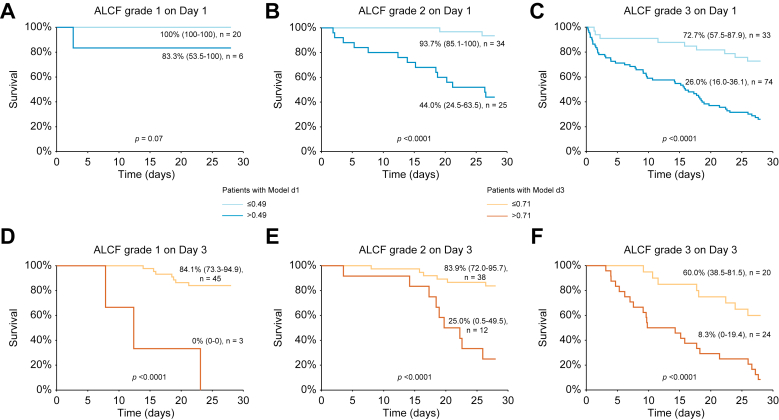
28-day Kaplan–Meier survival analysis of the overall Lausanne cohort according to the Model d1 (cut-off ≤ 0.49) and acute-on-chronic liver failure (ACLF) (A) grade 1, (B) grade 2, and (C) grade 3. The 28-day Kaplan–Meier survival analysis of the overall cohort according to the Model d3 (cut-off ≤0.71) and ACLF (D) grade 1, (E) grade 2, and (F) grade 3. The 28-day survival was estimated using the Kaplan–Meier method and compared with the log-rank test. Survival was expressed as a percentage with 95% CI.

To better evaluate the impact of our findings in clinical practice, we investigated the discriminative capacity of the two cut-offs with regards to the prediction of mortality throughout the different grades of ACLF. The 0.49 cut-off of Model d1 was able to discriminate two populations at low and high risk of mortality in patients with ACLF grade 2 (28-day survival rate of 93.7% [85.1–100] *vs.* 44.0% [24.5–63.5], *p* <0.0001) and grade 3 (28-day survival rate of 72.7% [57.5–87.9] *vs.* 26.0% [16.0–36.1], *p* <0.0001) but not in patients with ACLF grade 1 (28-day survival rate of 100% [100–100] *vs.* 83.3% [53.5–100], *p* = 0.07) (Fig. 2A–C). On Day 3, the cut-off of 0.71 was able to discriminate two populations at low and high risk of mortality in the three categories: ACLF grade 1 (28-day survival rate of 84.1% [73.3–94.9] *vs.* 0% [0–0], *p* <0.0001), ACLF grade 2 (28-day survival rate of 83.9% [72.0–95.7] *vs.* 25.0% [0.5–49.5], *p* <0.0001) and grade 3 (28-day survival rate of 60.0% [38.5–81.5] *vs.* 8.3% [0–19.4], *p* <0.0001) (Fig. 2D–F).

We then performed sensitivity analyses to evaluate the performance of Model d1 and Model d3 in male and female patients. In male patients, the AUROC of both scores outperformed CLIF-C ACLF-lactate on Day 1 (0.78 [0.68–0.85] *vs.* 0.69 [0.59–0.78], *p* = 0.002) and Day 3 (0.90 [0.83–0.94] *vs.* 0.86 [0.78–0.91], *p* = 0.04). By contrast, the AUROCs of these scores did not differ compared with CLIF-C ACLF-lactate on Day 1 (0.91 [0.79–0.96] *vs.* 0.90 [0.75–0.95], *p* = 0.62) and Day 3 (0.93 [0.81–0.97] *vs.* 0.93 [0.81–0.98], *p* = 0.91). In both male and female patients, Model d1 and Model d3 cut-offs were able to identify two populations at low and high risk of mortality at day 28 (Figs S2 and S3).

### Comparison of patients with and without sarcopenia in the Lausanne exploratory cohort

In a final step, to explain the poorer outcome observed in patients with sarcopenia, we investigated whether these patients had specific characteristics at baseline and during the ICU stay that distinguished them from non-sarcopenic patients. Regarding body composition, patients with sarcopenia had lower L3SMI (38.9 cm^2^/m^2^ [33.9–45.5] *vs.* 52.8 [48.2–57.8], *p* <0.0001), SMRA (35.0 HU [31.0–39.2] *vs.* 38.0 HU [32.0–43.0], *p* = 0.04), as well as VATI (36.8 cm^2^/m^2^ [21.2–62.4] *vs.* 55.1 cm^2^/m^2^ [35.7–86.5], *p* = 0.002), SATI (40.7 cm^2^/m^2^ [19.4–65.7] *vs.* 56.7 cm^2^/m^2^ [39.8–89.0], *p* = 0.02) and a greater VAT-RA (-79.6 HU [-85.5 to -75.3] *vs.* -82.8 cm^2^/m^2^ HU [-90.9 to -76.2], *p* = 0.02), as expected. Except for BMI (24.3 [kg/m^2^ 23.0–31.1] *vs.* 28.9 kg/m^2^ [21.7–31.4], *p* = 0.002), none of the clinical and biological characteristics or the severity scores at admission differed between patients with and without sarcopenia on Day 1 (Table 4). On Day 3, all severity scores, including MELD (22.9 [14.5–29.6] *vs.* 17.2 [11.6–26.2], *p* = 0.04), CLIF-C ACLF (66.1 [60.1–72.8] *vs.* 61.1 [55.8–69.2], *p* = 0.02, CLIF-C ACLF-lactate (66.3 [60.2–78.9] *vs.* 59.2 [53.2–66.6], *p* = 0.0003 and Model d3 (0.57 [0.34–0.89] *vs.* 0.15 [0.07–0.35], *p* <0.0001) were increased in patients with sarcopenia, indicating a worse course of organ failure in this population. When investigating the differences during ICU stay between the two populations, we observed that patients with sarcopenia more often required the use of RRT (30.5% *vs.* 15.5%, *p* = 0.05) and were more likely to develop invasive fungal infections (7.4% *vs.* 1.4%, *p* = 0.05). However, none of the other variables collected during the ICU stay, including the need for other organ support, development of hepatic encephalopathy, as well as the incidence and site of bacterial infections, differed between patients with and without sarcopenia (Table 5).

**Table 4:** **Comparison of baseline characteristics between patients with (n** = **121) and without (n** = **71) sarcopenia from the Lausanne cohort**.

	Non-sarcopenic (n = 71)	Sarcopenic (n = 121)	*p* value
Characteristics			
Age (years)	62.0 (54.0–72.0)	63.0 (53–70)	0.91
Sex (male)	49 (69.0)	92 (76.0)	0.33
Body mass index (kg/m^2^)	28.9 (21.7–31.4)	24.3 (23.0–31.1)	0.002
Ethnicity			0.45
Caucasian	61 (85.9)	100 (82.6)	
Hispanic	6 (8.5)	10 (8.3)	
Other	4 (5.6)	11 (9.1)	
Clinical frailty score	4.0 (3.0–4.0)	4.0 (4.0–4.5)	0.34
Aetiology			
Alcohol	43 (60.6)	86 (71.1)	0.26
Viral	14 (19.7)	20 (16.5)	
Metabolic	10 (14.1)	8 (6.6)	
Other	4 (5.6)	7 (5.8)	
Cause for ICU admission			
Sepsis	28 (39.4)	51 (42.1)	0.22
Bleeding	26 (36.6)	37 (30.6)	
Other	17 (23.9)	33 (27.3)	
Laboratory			
Leukocytes (G/L)	15.3 (10.4–19.5)	14.9 (9.9–21.6)	0.81
International normalised ratio	1.4 (1.3–1.7)	1.6 (1.3–2.0)	0.33
Bilirubin (mg/dl)	4.8 (3.7–7.9)	5.5 (3.7–8.7)	0.82
Aspartate aminotransferase (IU)	122.0 (53.0–426.5)	80.5 (47.0–230.25)	0.48
Albumin (g/L)	27.0 (24.0–33.0)	27.5 (23.0–31.0)	0.26
Creatinine (mg/dl)	1.3 (0.8–2.2)	1.5 (1.0–2.1)	0.77
Sodium (mmol/L)	139.0 (134.0–142.0)	137.0 (134.0–141.0)	0.83
Lactate (mmol/L)	3.3 (2.3–5.9)	4.5 (2.4–7.8)	0.36
Ammonia (μmol/L)	76.5 (57.0–106.5)	65 (51–112)	0.62
C-reactive protein (mg/L)	47.0 (14 0.0–112.0)	68.0 (24.0–116.0)	0.86
Organ failure			
Liver	17 (23.9)	35 (28.9)	0.21
Kidney	24 (33.8)	39 (32.2)	0.88
Brain	20 (28.2)	38 (25.6)	0.66
Coagulation	11 (15.5)	26 (21.5)	0.31
Circulation	61 (85.9)	107 (88.4)	0.61
Lung	36 (50.7)	58 (47.9)	0.72
Organ support			
Renal replacement therapy Vasopressors Mechanical ventilation	10 (14.1) 61 (85.9) 43 (60.6)	14 (11.6) 107 (88.4) 61 (50.4)	0.62 0.62 0.12
ACLF grade			
1 2 3	11 (15.5) 23 (32.4) 37 (52.1)	15 (12.4) 36 (29.8) 70 (57.8)	0.72
Scores			
MELD, on Day 1	21.2 (13.9–27.2)	22.7 (13.4–28.2)	0.32
CLIF-C ACLF, on Day 1	67.6 (61.4–71.8)	67.1 (61.8–73.4)	0.82
CLIF-C ACLF lactate, on Day 1	70.7 (60.9–79.9)	71.6 (65.2–80.8)	0.64
CLIF-C ACLF-lactate-sarcopenia (Model d1), on Day 1	0.24 (0.12–0.43)	0.62 (0.47–0.78)	<0.0001
MELD, on Day 3	17.2 (11.6–26.2)	22.9 (14.5–29.6)	0.04
CLIF-C ACLF, on Day 3	61.1 (55.8–69.2)	66.1 (60.1–72.8)	0.02
CLIF-C ACLF lactate, on Day 3	59.2 (53.2–66.6)	66.3 (60.2–78.9)	0.0003
CLIF-C ACLF-lactate-sarcopenia (Model d3), on Day 3	0.15 (0.07–0.35)	0.57 (0.34–0.89)	<0.0001
Body composition parameters			
L3SMI (cm^2^/m^2^)	52.8 (48.2–57.8)	38.9 (33.9–45.5)	<0.0001
Sarcopenia according to L3SMI sex-specific cut-offs	0 (0.0)	121 (100)	<0.0001
SMRA (HU)	38.0 (32.0–43.0)	35.0 (31.0–39.2)	0.04
IMAT (cm^2^/m^2^)	6.5 (4.5–11.4)	6.0 (3.5–9.6)	0.06
VAT (cm^2^/m^2^)	55.1 (35.7–86.5)	36.8 (21.2–62.4)	0.002
SAT (cm^2^/m^2^)	56.7 (39.8–80.9)	40.7 (19.4–65.7)	0.02
VSR	0.8 (0.5–1.4)	0.9 (0.6–1.4)	0.82
VAT-RA (HU)	-82.8 (-90.9 to -76.2)	-79.6 (-85.5 to -75.3)	0.02
SAT-RA (HU)	-90.9 (-98.1 to -77.1)	-84.9 (-93.4 to -76.6)	0.12

Continuous and categorical variables expressed in median (IQR) and n (percentages), respectively. Comparisons were performed using the Student’s t test or Mann-Whitney *U* test for quantitative variables or Chi-square and Fisher exact tests for categorical variables as appropriate.

ACLF, acute-on-chronic liver failure; CLIF-C, chronic liver failure consortium; HU, Hounsfield unit; ICU, intensive care unit; IMATI, intermuscular adipose tissue area index; MELD, Model for End-Stage Liver Disease; OR, odds ratio; SATI, subcutaneous adipose tissue area index; SMRA, skeletal muscle radiation attenuation; SAT-RA, subcutaneous adipose tissue area radiation attenuation; VAT-RA, visceral adipose tissue area radiation attenuation; VATI, visceral adipose tissue area index; VSR, visceral-on-subcutaneous adipose tissue area ratio.

**Table 5:** **Comparison of ICU stay characteristics between patients with (n** = **121) and without (n** = **71) sarcopenia from the Lausanne cohort**.

ICU stay characteristics	Non-sarcopenic (n = 71)	Sarcopenic (n = 121)	*p* value
Organ support characteristics			
Vasopressors during ICU stay, n (%)	69 (97.7)	117 (96.7)	0.81
Vasopressors duration, days	3.0 (2.0–6.0)	3.0 (2.0–7.5)	0.92
Mechanical ventilation during ICU stay, n (%)	55 (77.5)	82 (67.8)	0.21
Mechanical ventilation duration, days	3.0 (1.0–12.0)	6.0 (1.0–12.0)	0.93
Renal replacement therapy during ICU stay, n (%)	11 (15.5)	37 (30.5)	0.05
Renal replacement therapy duration, days	6.0 (2.0–12.0)	5.0 (2.0–13.0)	0.58
Hepatic encephalopathy during ICU stay, n (%)	66 (92)	102 (85)	0.23
Hepatic encephalopathy duration, days	4.5 (1.0–12.0)	5.0 (1.0–12.5)	0.92
Infectious events			
Bacterial, total, n (%)	54 (76.1)	100 (82.6)	0.32
Bacterial with documentation, n (%)	36 (66.7)	70 (70)	0.32
Probable fungal invasive infection, n (%)	1 (1.4)	9 (7.4)	0.05
Proven fungal invasive infection, n (%)	0 (0)	7 (5.7)	0.03
Infectious events according to site			
Pulmonary, n (%) Urinary tract, n (%) Spontaneous bacterial peritonitis, n (%) Others, n (%)	30 (53.6) 2 (3.6) 15 (26.8) 9 (16.1)	48 (47.1) 10 (9.8) 23 (22.6) 21 (20.6)	0.43
Nutritional characteristics			
Total duration, days	7.0 (4.0–20.0)	8.0 (3.0–19.0)	0.82
Support quantity, kcal/day	825.0 (335.3–1409.0)	972.0 (397.3–1312.0)	0.62
Bedsores, n (%)	8 (11.3)	23 (19.0)	0.28

Continuous and categorical variables expressed in median (interquartile range) and n (percentages), respectively. Comparisons were performed using the Student’s *t* test or Mann-Whitney *U* test for quantitative variables or Chi-square and Fisher exact tests for categorical variables as appropriate.

### Evaluation of the models in the Villejuif external cohort

Fifty-eight of the 200 (29%) patients critically ill with cirrhosis with ACLF admitted to the liver ICU of Paul Brousse University Hospital underwent a CT within the set timeframe from admission, allowing for body composition assessment, and were included in the analyses. Comparison of the main characteristics between patients from the exploratory and this cohort are provided in Table S7.

The median interval between CT and admission was -1 day (-3 to +1 days). Forty-six patients were male (79.3%). Eight patients (10.3%) underwent LT before Day 28 and 41 patients (70.7%) had sarcopenia. Compared with the Lausanne cohort, patients from this cohort were younger (55.3 years [48.7–62.9] *vs.* 62.0 years [53.2–70.0], *p* = 0.0002) and more severely ill, as illustrated by higher MELD (31.0 [25.0–37.0] *vs.* 21.9 [15.1–27.9], *p* <0.0001), CLIF-C ACLF (77.5 [73.5–82.9] *vs.* 67.5 [51.8–72.7], *p* <0.0001) and CLIF-C ACLF lactate scores (78.9 [72.2–87.8] *vs.* 71.5 [64.1–80.2], *p* <0.0001).

In this cohort, 28-day survival was lower in patients with compared with those without sarcopenia (31.7% [17.5–46.0] *vs.* 64.7% [42.0–87.5], *p* = 0.02).

**Fig. 3:**
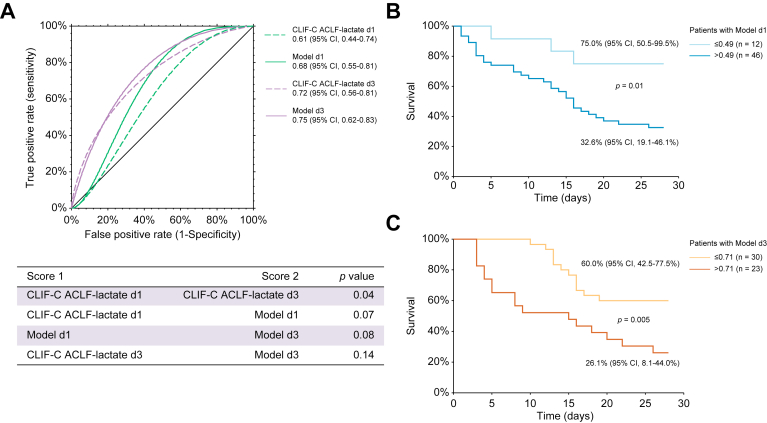
Performance of the available and newly developed models (Model d1 and d3) in the external Villejuif cohort. (A) Receiver operating characteristic curves for survival at 28 days in the overall cohort as determined by the Chronic Liver Failure Consortium (CLIF-C) acute-on-chronic liver failure (ACLF)-lactate-sarcopenia score at Day 1 (Model d1, 0.68 [95% CI 0.55–0.81]) and Day 3 (Model d3, 0.75 [95% CI 0.62–0.83]) *vs.* the CLIF-C ACLF-lactate score at Day 1 (0.61 [95%CI 0.44–0.74], *p* = 0.07) and Day 3 (0.72 [95% CI 0.56-0.81], *p* = 0.14). (B) The 28-day Kaplan–Meier survival analysis of the overall cohort according to the CLIF-C ACLF-lactate-sarcopenia score at Day 1 (Model d1, cut-off ≤0.49). (C) The 28-day Kaplan–Meier survival analysis of the overall cohort according to the CLIF-C ACLF-lactate-sarcopenia score at Day 3 (Model d3, cut-off ≤0.71). The 28-day survival was estimated using the Kaplan–Meier method and compared with the log-rank test. Survival was expressed as a percentage with 95% CI. The differences in terms of diagnostic accuracy between the models and the CLIF-C ACLF-lactate score on Days 1 and 3 were assessed by comparison of area under the receiver operating characteristic curves using the z test described by Zhou *et al.*^39^

We then evaluated the performance of the newly developed Model d1 and d3 in this cohort. For these two models, the Hosmer–Lemeshow Chi-square statistic was 5.9 (8 df, *p* = 0.66) and 8.3 (7 df, *p* = 0.31), respectively, confirming similar observed and predicted 28-day mortality rate across 10 stratified groups. Overall, 69.0% and 71.1% of patients were correctly classified with Model d1 and Model d3, respectively. The AUROCs for 28-day survival probability (Fig. 3A) were 0.68 (95% CI 0.55–0.81) and 0.75 (95% CI 0.62–0.83), respectively. There was a trend toward greater AUROCs of Model d1 and d3 compared with CLIF-C ACLF-lactate score on Day 1 (0.61 [0.44–0.74], *p* = 0.07) and Day 3 (0.72 [0.56–0.81], *p* = 0.14) (Fig. 3A). In this cohort, patients with a Model d1 ≤0.49 had a 28-day survival rate of 75.0% (95% CI 50.5–99.5) *vs.* 32.6% (95% CI 19.1–46.2) for those with a score >0.49 (*p* = 0.01) (Fig. 3B). In parallel, patients with a Model d3 ≤0.71 had a 28-day survival rate of 60.0% (95% CI 42.5–77.5) *vs.* 26.1% (95% CI 8.1–44.0) (*p* = 0.005) (Fig. 3C).

We then performed sensitivity analyses in male and female patients. In male patients, there was a trend toward greater AUROCs of Model d1 and d3 compared with CLIF-C ACLF-lactate on Day 1 (0.67 [0.49–0.81] *vs.* 0.61 [0.38–0.77], *p* = 0.08) and Day 3 (0.74 [0.54–0.86] *vs.* 0.71 [0.52–0.82], *p* = 0.20), whereas, in female patients, no trends was observed on Day 1 (0.69 [0.33–0.88] *vs.* 0.64 [0.30–0.84], *p* = 0.83) and Day 3 (0.85 [0.46–0.97] *vs.* 0.87 [0.42–0.98], *p* = 0.40).

## Discussion

In the present study, we investigated whether modifications of body composition measured by CT in patients acutely ill with cirrhosis with ACLF had an independent impact on short-term survival. Eight variables of body composition were explored, including muscle and adipose tissue areas and densities. We observed that sarcopenia, assessed by L3SMI, was the only variable independently associated with 28-day survival in our cohort, together with specific organ failure scores of ACLF on Days 1 and 3. However, in sex-specific analyses, this association was only observed in male patients. To further progress the predictive performance of the available scores, we included sarcopenia into the CLIF-C ACLF-lactate score on Days 1 and 3. The newly developed models (Models d1 and d3) allowed for improvement of the prediction of short-term mortality, especially in male patients, in whom rapid decision-making processes are key and can be influenced by prognostic scores. Comparable results regarding the sex specificity of the performance of sarcopenia and the newly developed models were observed in an external cohort of in patients critically ill with cirrhosis with ACLF. Compared with patients without sarcopenia, those with sarcopenia showed a poorer course of OF during the first 3 days. They were also more likely to develop invasive fungal infections and to require RRT.

Frailty and sarcopenia are two phenotypic expressions of malnutrition.^40^ Their negative impact in patients with decompensated cirrhosis, LT candidates, and LT recipients has been documented extensively.^12^^,^^18^^,^^28^^,^[41], [42], [43] Frailty is well defined in patients with decompensated cirrhosis.^40^ However, its precise assessment can be difficult or impossible in patients acutely ill with ACLF because of the use of sedation or mechanical ventilation or the presence of severe hepatic encephalopathy.^12^^,^^28^^,^^44^ By contrast, in this specific situation, sarcopenia could easily and readily be evaluated by the L3SMI on CT because they are regularly performed to search for intra-abdominal complications (infection or portal vein thrombosis) and/or hepatocellular carcinoma. In the present study, we observed that 30–44% of all patients admitted to the ICU with ACLF underwent a CT (from -5 to +2 days after admission).

Besides sarcopenia, there is growing evidence for an impact of body composition on outcomes in liver diseases, especially evaluation of adipose tissue density. Adipose tissue density could serve as a surrogate marker of adipose tissue quality, which is influenced by various tissue components (e.g. not only water and blood flow, but also adipocyte size and lipid content). In hypercatabolic conditions, adipose tissue provides energy, stimulates insulin responses, glucose, and lipid metabolism, and impacts immune responses through release of adipokines.^27^ Consequently, a modification of its density could result in adipose tissue dysfunction.

In the present study, we investigated whether one or several body composition parameters measured by CT were independently associated with short-term mortality in patients critically ill with ACLF. To this end, we used a recently developed deep learning-based tool offering the possibility to acquire multiple body composition parameters within a short timeframe. Considering the available evidence linking modifications of body composition to outcomes not only in liver disease, but also in cancer, diabetes, chronic kidney disease, and inflammatory bowel disease,[45], [46], [47] we believe that tertiary centres should develop dedicated programs for body composition evaluation in acute and chronic disease.

Whereas, in the overall Lausanne cohort and in male patients, sarcopenia assessed by L3SMI was the only predictive independent factor associated with 28-day mortality, this was not the case in the female patients. In the latter, the results of logistic regression suggested that SAT-RA could be more strongly associated with the outcome compared with muscle parameters. As suggested by others,^27^ adipose tissue atrophy could be increasingly observed in female patients in response to the catabolic stress of chronic disease, whereas males primarily use other energy sources, such as muscle, in hypercatabolic states. This might explain why, in female patients, according to AUROC comparisons, the performance of the newly developed scores did not improve the prediction ability of CLIF-C ACLF-lactate.

In male patients, Model d1 and d3 outperformed the CLIF-C ACLF-lactate score on Days 0 and 3, with the cut-offs allowing discrimination between ACLF grade 2 and 3 patients with more favourable or poorer outcomes on Days 1 and 3. Despite a limited sample size, the sex specificity of these findings was also supported by the external cohort from Villejuif. Hence, the newly developed models could be used to assist the management of male patients, who in general account for 70–80% of the population critically ill with ACLF. In ACLF, patient management relies on the treatment of the precipitating event and the use of organ support. In selected patients with persistent OF, LT is the only therapeutic option.^1^ Accordingly, in the case of eligibility for LT, identification of patients with a worse course is important to seize the narrow window of opportunity. In the male population, the cut-offs of Model d1 and d3 could assist identification of these patients to establish a swift LT evaluation and listing. By contrast, in the case of non-eligibility for LT, rapid identification of male patients with a very high risk of mortality can help shift discussion toward a limitation of care. However, a decision to limit care is irrevocable in most instances in this situation and low survival probabilities might be perceived differently by caregivers and patient family members. Balancing these aspects while maintaining a patient's life, dignity, and wishes is fraught with statistical and ethical difficulties, and a single score is an unlikely final referee.^48^ This is especially true when the performance of the score is not consistent from one cohort to another. The CLIF-C ACLF score has been proposed to identify a threshold for the futility of care.^49^ However, the performance of the CLIF-C ACLF score has also been reported to be good but not excellent in external cohorts^8^ and as observed here. The performance of the CLIF-C ACLF score might be influenced by the expertise of the centre, approaches regarding withdrawal of care, as well as access to LT for patients with a severe course of ACLF. In this context, sarcopenia assessed by L3SMI could allow for better assessment of male patient condition across different cohorts and might be a more consistent tool to guide a discussion of limitation of care in patients with the highest risk of death. The present study also suggests that sarcopenia improves prognosis prediction to a greater extent on Day 1 compared with Day 3. Such observation recalls the weight of the course of OF in the setting of ACLF on short-term outcomes and the need for sequential reassessment over the ICU stay.

Another finding of the present study is the greater risk of developing an invasive fungal infection in patients with ACLF and sarcopenia. The occurrence of invasive fungal infection is always associated with a severely immunocompromised state. Muscle mass correlates with impairment of metabolic resources that are crucial to mitigate the intense systemic inflammation associated with the immune dysfunction observed in patients with cirrhosis and ACLF. Immune exhaustion is one of the main possible consequences of the deficient metabolic resources observed in patients with sarcopenia, which could explain the higher risk of sepsis-related mortality.^50^^,^^51^ Therefore, in these patients, we recommend screening regularly for the occurrence of invasive fungal infection and treating with antifungal medication any new sepsis in the absence of a positive bacterial examination.

It is well established that body composition is sex and ethnicity specific. Hence, the limited sample size of female patients in both cohorts as well as the dominant White ethnicity of patients are two of the main limitations of our study. This specifically prevents us from extrapolating our results to all patients with ACLF admitted to the ICU in other regions of the world. In future studies, there is a need to include a larger sample size of female patients. In particular, it would be important to definitively rule out the association between sarcopenia and short-term outcome in female patients as well as to investigate whether SAT-RA could help identifying those with lower survival chances. In both cohorts, it is worth noticing the excellent discrimination performance of CLIF-C ACLF-lactate on Day 3 in female patients. This performance could partly explain why body composition parameters were not independently associated with short-term outcome. Whether ACLF is less dynamic in females compared with males remains to be explored because it could impact the clinical management of patients.

Body composition can change rapidly in the days preceding and following ICU admission. In the Lausanne cohort, 90% of CTs were performed between 5 days before and 2 days after admission, with a median of 0 days (IQR, -2 to +1 days). Therefore, we consider that the time frame of evaluation of body composition in the exploratory Lausanne cohort was optimal. Accordingly, we narrowed the timeframe of the delay between admission and CT to -5 to +2 days.

In addition, the present study raises several questions that its retrospective nature could not address. In particular, it would be interesting to study whether patients with sarcopenia have a specific catabolic profile that could be optimised by early and dedicated nutritional support. Guidelines for clinical nutrition in the ICU supported by numerous studies have suggested that nutritional support should not be introduced before 48 h to avoid overfeeding and refeeding.^52^ However, nutritional support use in patients with sarcopenia showing severe catabolic conditions and immune exhaustion could have beneficial effects on the balance between protein synthesis and catabolism, as well as a favourable impact on immune dysfunction. Accordingly, a cautious evaluation of the early introduction of nutritional support could a focus of future prospective studies in the field.

In patients critically ill with cirrhosis with ACLF in our overall cohort and in male patients, among the different parameters of body composition evaluated by CT, sarcopenia assessed by L3SMI was the only variable independently associated with 28-day survival. However, the inclusion of sarcopenia in the CLIF-C ACLF lactate score on Days 1 and 3 improved prognostication only for male patients, highlighting a sex specificity for this parameter. The use of these scores could facilitate clinical decision making. Over the first 3 days of ICU management, patients with sarcopenia had a poorer course of OF associated with increased use of RRT and an increased risk of invasive fungal infection. Future prospective studies should explore the immune cell metabolism and function in patients with sarcopenia and evaluate the potential benefit of early nutritional support in this population. In female patients, none of the body composition parameters were independently associated with 28-day survival and further research is needed to investigate in particular the association between SAT-RA and short-term outcomes in this population.

## Financial support

The authors received no financial support to produce this manuscript.

## Authors’ contributions

Study design: T.M., J.V., D.M., F.B., F.A.

Data acquisition: T.M., S.C.S., J.V., P.I., A.D., A.S., A.C., A.W., J.K., D.M., F.B., F.A.

Statistical analysis: F.A.

Drafting of manuscript and critical review: all authors.

## Data availability statement

Detailed data are not openly available (reasons of sensitivity – human data) and are available from the corresponding authors upon reasonable request.

## Conflicts of interests

None of the contributing authors has any disclosures related to this work.

Please refer to the accompanying ICMJE disclosure forms for further details.
