# Supplementary material for: Body composition and short-term mortality in patients critically ill with acute-on-chronic liver failure
Source: JHEP Rep. 2023 Apr 7;5(8):100758. doi: 10.1016/j.jhepr.2023.100758 (PMC10403365; doi:10.1016/j.jhepr.2023.100758)
Supplement: Multimedia component 1 [file mmc1.pdf]

Journal of Hepatology, Volume ■

Supplemental information

Body composition and short-term mortality in patients critically ill with acute-on-chronic liver failure

Thomas Mangana del Rio, Sophie-Caroline Sacleux, Julien Vionnet, Philippe Ichaï, Alban Denys, Antoine Schneider, Audrey Coilly, Montserrat Fraga, Alexandre Wetzel, Joachim Koerfer, Jean-Daniel Chiche, Faouzi Saliba, Darius Moradpour, Fabio Becce, and Florent Artru

Supplementary materials

Body composition and short-term mortality in critically ill patients with acute on chronic liver failure

Thomas Mangana del Rio, Sophie-Caroline Sacleux, Julien Vionnet, Philippe Ichaï, Alban Denys, Antoine Schneider, Audrey Coilly, Montserrat Fraga, Alexandre Wetzel, Joachim Koerfer, Jean-Daniel Chiche, Faouzi Saliba, Darius Moradpour, Fabio Becce and Florent Artru

Table of contents

Patients and Methods…………………………………………………………………..…..page 2
Figures……………………………………………………………………………………..page 3
Tables………………………………………………………………………………………page 6

Patients and Methods

1. ICD-10 codes

The following ICD-10 codes were used to retrospectively screen for the presence of liver cirrhosis:

- chronic liver disease: K70.0, K70.2, K73.X, K754, K758, K75.9, K76.0, B18.0, B18.1, B18.2, B18.8, B18.9
- cirrhosis codes: K70.30, K70.31, K71.7, K72.1, K74.4, K74.5, K74.60, K74.69, K74.3, K72.1, K72.9
- alcoholic liver disease: K70.4, K70.9
- ascites and spontaneous bacterial peritonitis: R18.8, K70.31, K70.11 K65.2, K65.0, K65.9
- varices: I85.9, I85.00, I86.4, I98.2, I85.10 I85.01, I98.3, I85.11
- hepatic encephalopathy: K70.41, K72.11, K72.91, B15.0, B16.0, B16.2, 17.11, B19.0, B19.11, B19.21, G31.2, G93.4
- portal hypertension and hepatorenal syndrome: K76.6, K76.7

2. Definition and site of infection

Diagnostic criteria for bacterial infections were the following:

- spontaneous bacterial peritonitis: polymorphonuclear (PMN) cell count in ascitic fluid ≥ 250/mm3;
- urinary tract infection: abnormal urinary sediment (> 10 leukocytes/field) and positive urinary culture;
- bacteraemia: positive blood cultures;
- pneumonia: clinical signs of infection and new infiltrates on chest x-ray;
- skin and soft tissue infections: clinical signs of infection, swelling, erythema, heat and tenderness in the skin;
- cholangitis: cholestasis, right upper quadrant pain and/or jaundice and radiological evidence for biliary obstruction;
- secondary peritonitis: PMN count in ascitic fluid ≥ 250/mm³ and evidence (abdominal CT/surgery) of an intraabdominal source of infection;
- *Clostridioides difficile* infection: positive stool toxin in a patient with diarrhea.

The other infections were diagnosed according to conventional criteria.

Fig. S1. Flowchart of patient inclusion from the Lausanne cohort. ACLF, acute-on-chronic liver failure; ICU, intensive care unit.

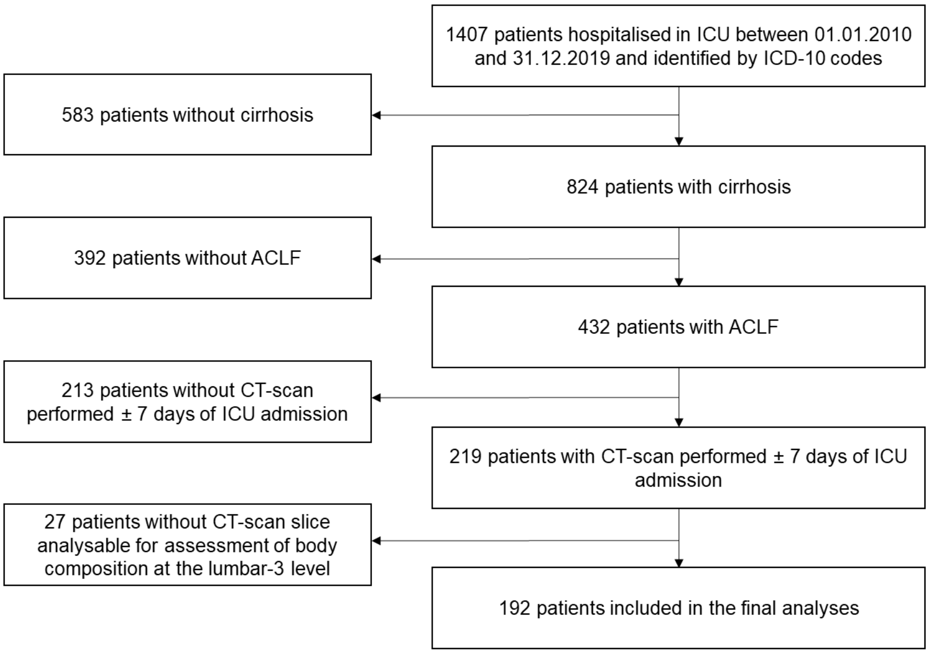

Mangana del Rio *et al*. Supplementary Figure 1

Fig. S2. Performances of the available and newly developed models (Model d1 and d3) in male patients from the Lausanne cohort. (**A**) Receiver operating characteristic curves for survival at 28 days in the male patients' cohort as determined by the Model d1 (0.78 [0.68-0.85]) and Model d3 (0.90 [0.83-0.94]) *vs*. the CLIF-C ACLF-lactate score on day 1 (0.69 [0.59-0.78], p=0.002) and day 3 (0.86 [0.78-0.91], p=0.04). **(B)** Kaplan-Meier survival analysis of the male patients' cohort according to the Model d1 (cut-off ≤ 0.49). **(C)** Kaplan-Meier survival analysis of the male patients' cohort according to the Model d3 (cut-off ≤ 0.71).

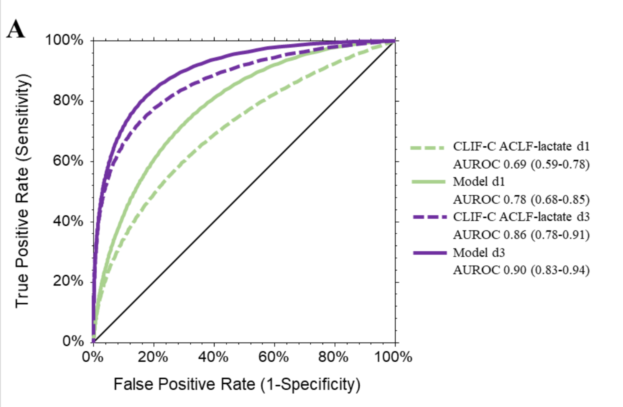

Score 1	Score 2	p value
CLIF-C ACLF-lactate d1	CLIF-C ACLF-lactate d3	0.0002
CLIF-C ACLF-lactate d1	Model d1	0.002
Model d1	Model d3	0.002
CLIF-C ACLF-lactate d3	Model d3	0.04

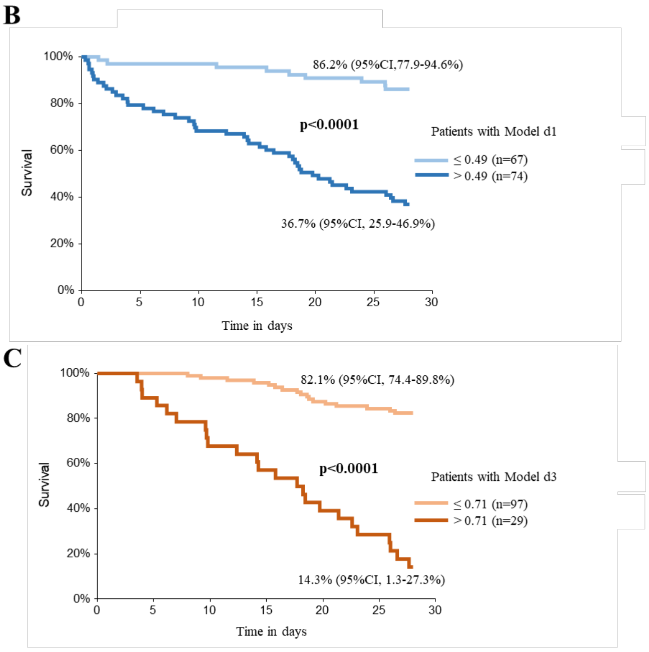

Fig. S3: Performances of the available and newly developed models (Model d1 and d3) in female patients from the Lausanne cohort. (**A)** Receiver operating characteristic curves for survival at 28 days in female patients' cohort as determined by the Model d1 (0.91 [0.79-0.96]) and Model d3 (0.93 [0.81-0.97]) *vs*. the CLIF-C ACLF-lactate score on day 1 (0.90 [0.75-0.95], p=0.62) and day 3 (0.93 [0.81-0.98], p=0.91). **(B)** Kaplan-Meier survival analysis of the female patients' cohort according to the Model d1 (cut-off ≤ 0.49). **(C)** Kaplan-Meier survival analysis of the female patients' cohort according to the Model d3 (cut-off ≤ 0.71).

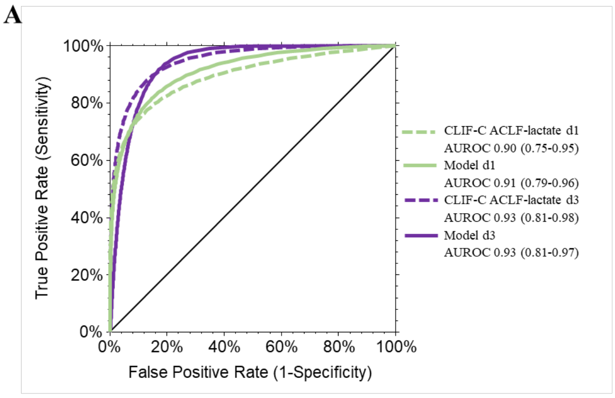

Score 1	Score 2	p value
CLIF-C ACLF-lactate d1	CLIF-C ACLF-lactate d3	0.17
CLIF-C ACLF-lactate d1	Model d1	0.62
Model d1	Model d3	0.18
CLIF-C ACLF-lactate d3	Model d3	0.91

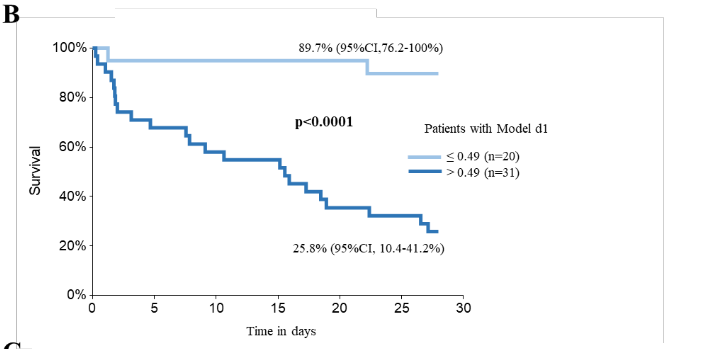

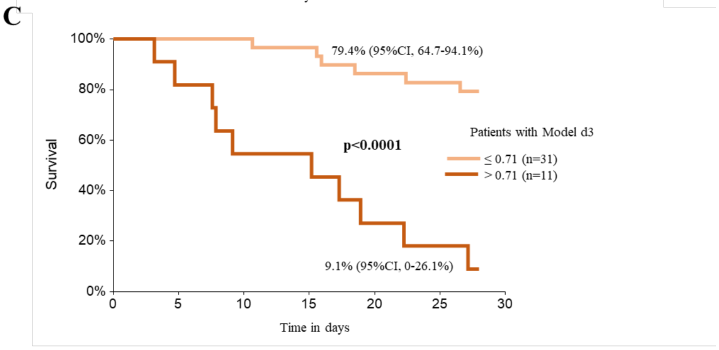

Mangana del Rio *et al*. Supplementary Figure 3

Table S1: Comparison of the main characteristics on day 1 between patients from Lausanne included in the final analyses (n=192) and patients with cirrhosis and ACLF not included in the final analyses (CT not performed or absence of CT-scan slice allowing for body composition evaluation) (n=240). Continuous and categorical variables are expressed respectively in median (interquartile range) and N (percentages). Comparisons were performed using the Student's t-test or Mann-Whitney U test for quantitative variables or Chi-Square and Fisher exact tests for categorical variables as appropriate

	**Overall population included in the final analyses (n=192)**	**Patients with cirrhosis and ACLF not included in the final analyses (CT not performed or absence of CT-scan slice allowing for body composition evaluation) (n=240))**	**p**
**Characteristics**			
Age (years)	62.0 (53.2-70.0)	63.0 (54.0-70.8)	0.72
Sex (male)	141 (73.5)	181 (76.7)	0.45
BMI (kg/m^2)	25.8 (22.3-31.2)	26.1 (22.7 – 28.8)	0.36
Ethnicity<br>Caucasian<br>Hispanic<br>Other	 161 (83.8)<br>16 (8.3)<br>15 (7.8)	 178 (74.2)<br>34 (14.2)<br>28 (11.7)	0.09
**Aetiology**			
Alcohol<br>Viral<br>Metabolic<br>Other	129 (67.2)<br>34 (17.7)<br>18 (9.4)<br>11 (5.7)	167 (70.7)<br>35 (14.8)<br>20 (8.5)<br>14 (6.0)	0.96
**Cause for ICU admission**			
Sepsis<br>Bleeding<br>Other	79 (41.1)<br>63 (32.8)<br>50 (26.1)	82 (34.2)<br>94 (39.1)<br>64 (26.7)	0.22
**Laboratory on day 1**			
Leukocytes (G/l)	15.0 (10.1-20.8)	13.6 (9.7-19.3)	0.04
INR	1.5 (1.3-1.8)	1.4 (1.2-1.7)	0.07
Bilirubin (mg/dl)	5.3 (3.7-8.2)	4.5 (3.3-6.6)	0.32
AST (IU)	93.0 (48.0-299.5)	80 (44.0-268.0)	0.31
Albumin (g/l)	27.0 (23.2-31.0)	28.0 (24.0-32.0)	0.26
Creatinine (mg/dl)	1.4 (1.0-2.1)	1.4 (0.8-2.3)	0.28
Sodium (mmol/l)	138.0 (134.0-141.0)	138.0 (134.0-141.0)	0.83
Lactate (mmol/l)	4.1 (2.4-7.4)	3.0 (2.0-5.4)	0.21
Ammonia, (µmol/l)	71.0 (53.0-112.0)	65.0 (45-101)	0.47
CRP (mg/l)	55.0 (20.0-112.5)	39.0 (13.0-92.0)	0.02
**ACLF grade on day 1**			
0<br>1<br>2<br>3	0 (0)<br>26 (13.6)<br>59 (30.7)<br>107 (55.7)	0 (0)<br>22 (9.1)<br>95 (39.6)<br>123 (51.3)	0.09
**Scores on day 1**			
MELD	21.9 (15.1-27.9)	19.1 (14.4-28.6)	0.30
CLIF-C ACLF	67.5 (51.8-72.7)	66.6 (61.5-72.1)	0.64
CLIF-C ACLF-lactate	71.5 (64.1-80.2)	69.5 (62.7-78.3)	0.08

Table S2: Comparison of the main characteristics on day 1 between patients included in the final analyses (n=192) and patients with cirrhosis and without ACLF in whom a CT was performed at admission to the intensive care unit (ICU) ± 7 days (n=131). Continuous and categorical variables are expressed in median (interquartile range) and N (percentages), respectively. Comparisons were performed using the Student's t-test or Mann-Whitney U test for quantitative variables or Chi-Square and Fisher exact tests for categorical variables as appropriate

	Overall population included in the final analyses (n=192)	Cirrhotic patients without ACLF who underwent a CT on admission ± 7 days (n=131)	p
**Characteristics**			
Age (years)	62.0 (53.2-70.0)	66.0 (50.0-75.0)	0.89
Sex (male)	141 (73.5)	93 (70.9)	0.24
BMI (kg/m^2)	25.8 (22.3-31.2)	25.4 (21.4 – 27.6)	0.58
Ethnicity<br>Causasian<br>Hispanic<br>Other	 161 (83.8)<br>16 (8.3)<br>15 (7.8)	 102 (77.9)<br>13 (9.9)<br>14 (10.7)	0.18
Clinical frailty scale (score)	4.0 (3.0-4.0)	3.0 (2.0-4.0)	0.02
**Aetiology**			
Alcohol<br>Viral<br>Metabolic<br>Other	129 (67.2)<br>34 (17.7)<br>18 (9.4)<br>11 (5.7)	99 (75.6)<br>6 (4.5)<br>20 (15.4)<br>6 (4.5)	0.007
**Cause for ICU admission**			
Sepsis<br>Bleeding<br>Other	79 (41.1)<br>63 (32.8)<br>50 (26.1)	10 (7.6)<br>58 (44.3)<br>63 (48.1)	<0.0001
**Laboratory on day 1**			
Leukocytes (G/l)	15.0 (10.1-20.8)	11.6 (7.2-16.7)	0.03
INR	1.5 (1.3-1.8)	1.3 (1.1-1.6)	0.009
Bilirubin (mg/dl)	5.3 (3.7-8.2)	4.2 (3.3-5.6)	0.001
AST (IU)	93.0 (48.0-299.5)	73 (34.0-168.0)	0.07
Albumin (g/l)	27.0 (23.2-31.0)	32.0 (27.0-38.0)	0.007
Creatinine (mg/dl)	1.4 (1.0-2.1)	1.1 (0.7-1.9)	0.03
Sodium (mmol/l)	138.0 (134.0-141.0)	138.0 (134.0-142.0)	0.93
Lactate (mmol/l)	4.1 (2.4-7.4)	2.4 (1.3-3.8)	0.21
Ammonia, (µmol/l)	71.0 (53.0-112.0)	61.0 (41-92)	0.47
CRP (mg/l)	55.0 (20.0-112.5)	45.0 (18.0-98.0)	0.07
**ACLF grade on day 1**			
0<br>1<br>2<br>3	0 (0)<br>26 (13.6)<br>59 (30.7)<br>107 (55.7)	131 (100)<br>0 (0)<br>0 (0)<br>0 (0)	<0.0001
**Scores on day 1**			
MELD	21.9 (15.1-27.9)	16.1 (12.4-21.6)	<0.0001
CLIF-C ACLF	67.5 (51.8-72.7)	46.8 (41.4-51.1)	<0.0001
CLIF-C ACLF-lactate	71.5 (64.1-80.2)	47.1 (42.1-52.3)	<0.0001
**Outcome**			
28-day survival, % (95%CI)	58.2 (51.2-65.2)	94.5 (90.1-98.5)	<0.0001
**Body composition parameters**			
L3SMI (cm^2/m^2)	43.2 (37.1-50.1)	46.8 (36.3-57.8)	0.009
Sarcopenia according to L3SMI sex specific cut-offs	121 (63.0)	61 (46.5)	0.01
SMRA (HU)	36.0 (31.0-41.0)	39.9 (28.6-47.2)	0.08
IMATI (cm^2/m^2)	6.3 (3.9-10.0)	4.9 (2.8-7.9)	0.01
VATI (cm^2/m^2)	44.3 (24.4-69.9)	49.4 (22.6-87.1)	0.03
SATI (cm^2/m^2)	48.6 (27.0-70.3)	53.0 (31.1-78.9)	0.01
VSR	0.9 (0.6-1.4)	0.9 (0.6-1.8)	0.18
VAT-RA (HU)	-81.2 (-88.5 to -75.8)	-84.5 (-92.8 to -78.8)	0.002
SAT-RA (HU)	-86.6 (-95.7 to -76.8)	-88.3 (-100.1 to -75.2)	0.02

Table S3: Univariable and multivariable analyses of predictors of 28-days mortality in male patients from Lausanne cohort on day 1 (n=141)

	Univariable analysis			Multivariable analysis		
**Covariant**	OR	95% CI	p value	OR	95% CI	p value
**Characteristics**						
Age (years)	0.99	0.96-1.02	0.6			
Sex (male)	-	-	-			
BMI (kg/m^2)	1.03	0.96-1.10	0.4			
Ethnicity						
Caucasian	-	-	-			
Hispanic	2.42	0.82-5.03	0.15			
Other	0.81	0.11-6.21	0.45			
Clinical frailty scale (score)	1.05	0.81-1.92	0.22			
**Aetiology**						
Alcohol	-	-	-	-	-	-
Viral	2.26	0.98-5.23	0.06	2.09	0.71-6.13	0.17
Metabolic	0.57	0.14-2.28	0.43	0.54	0.09-3.07	0.48
Other	0.34	0.04-3.09	0.34	0.29	0.03-3.02	0.30
**Cause for ICU admission**						
Sepsis	-	-	-	-	-	-
Bleeding	0.43	0.19-0.95	0.04	0.79	0.28-2.16	0.64
Other	0.59	0.23-1.49	0.26	0.81	0.22-2.95	0.75
**Laboratory**						
Leukocytes (G/l)	1.00	0.96-1.03	0.91			
INR	3.23	1.61-6.49	0.0009			
Bilirubin (mg/dl)	1.11	1.04-1.20	0.002			
AST (IU)	1.00	0.99-1.01	0.11			
Albumin (g/l)	0.98	0.89-1.02	0.24			
Creatinine (mg/dl)	1.46	1.05-2.04	0.02			
Sodium (mmol/l)	0.98	0.93-1.03	0.62			
Lactate (mmol/l)	1.15	1.08-1.24	0.0006			
Ammonia, (μmol/l)	1.00	0.99-1.01	0.67			
CRP (mg/l)	0.99	0.99-1.01	0.55			
**Organ failure**						
Liver	5.01	1.67-15.01	0.003			
Kidney	2.22	1.08-4.60	0.02			
Brain	1.06	0.92-1.89	0.16			
Coagulation	5.24	2.10-14.28	0.0005			
Circulation	4.71	1.03-21.80	0.02			
Lungs	1.63	1.03-3.22	0.02			
**Organ support**						
RRT	2.20	1.10-6.33	0.02			
Vasopressors	4.71	1.03-21.80	0.02			
Mechanical ventilation	4.11	1.33-12.75	0.01			
**Scores**						
MELD	1.08	1.04-1.13	<0.0001			
ACLF grade	3.43	1.85-6.34	<0.0001			
CLIF-C ACLF	1.07	1.03-1.12	0.001			
CLIF-C ACLF lactate	1.09	1.04-1.14	<0.0001	1.08	1.03-1.13	0.0003
**Body composition parameters**						
L3SMI (cm^2/m^2)*	0.95	0.91-0.98	0.03	0.97	0.94-1.01	0.07
Sarcopenia (L3SMI cut-offs)*	3.91	1.74-8.75	0.0009	3.21	1.13-9.05	0.03
SMRA (HU)	0.98	0.93-1.03	0.50			
IMATI (cm^2/m^2)	0.97	0.91-1.03	0.37			
VATI (cm^2/m^2)	0.99	0.98-1.01	0.35			
SATI (cm^2/m^2)	1.00	0.99-1.01	0.40			
VSR	0.61	0.33-1.26	0.21			
VAT-RA (HU) ¶	1.04	1.01-1.09	0.05	1.03	0.97-1.09	0.24
SAT-RA (HU) ¶	1.02	0.99-1.05	0.09	1.02	0.98-1.05	0.27
SAT-RA (HU), according to (27)	1.42	0.53-3.81	0.48			

*Not included in the multivariable analysis to avoid collinearity with sarcopenia according to L3SMI sex specific cut-offs.

¶ Not included in the multivariable analysis to collinearity (Spearman: 0.78 DF 102, $p<0.0001$).

Table S4: Univariable and multivariable analyses of predictors of 28-days mortality female patients from Lausanne cohort on day 1 (n=51)

	Univariable analysis			Multivariable analysis		
**Covariant**	OR	95% CI	p value	OR	95% CI	p value
**Characteristics**						
Age (years)	1.03	0.98-1.08	0.6			
Sex (male)	-	-	-			
BMI (kg/m^2)	1.00	0.89-1.12	0.9			
Ethnicity						
Caucasian	-	-	-			
Hispanic	4.58	0.27-9.33	0.23			
Other	0.89	0.21-8.12	0.67			
Clinical frailty score	1.08	0.63-2.20	0.61			
**Aetiology**						
Alcohol	-	-	-			
Viral	1.11	0.14-8.22	0.91			
Metabolic	1.11	0.19-6.290	0.89			
Other	1.67	0.24-11.26	0.59			
**Cause for ICU admission**						
Sepsis	-	-	-			
Bleeding	0.76	0.16-3.33	0.70			
Other	0.82	0.24-2.83	0.75			
**Laboratory**						
Leukocytes (G/l)	1.09	1.04-1.14	<0.0001			
INR	5.97	2.84-12.54	<0.0001			
Bilirubin (mg/dl)	1.08	1.03-1.13	0.0004			
AST (IU)	1.00	0.99-1.01	0.31			
Albumin (g/l)	1.07	1.00-1.15	0.03			
Creatinine (mg/dl)	1.16	0.70-1.95	0.55			
Sodium (mmol/l)	1.01	0.93-1.10	0.72			
Lactate (mmol/l)	1.16	1.02-1.32	0.02			
Ammonia, (µmol/l)	1.00	0.99-1.01	0.32			
CRP (mg/l)	1.00	0.99-1.01	0.89			
**Organ failure**						
Liver	2.28	1.02-13.76	0.04			
Kidney	1.76	0.87-5.57	0.09			
Brain	1.05	0.76-3.61	0.23			
Coagulation	1.64	1.03-6.04	0.03			
Circulation	4.23	0.78-22.84	0.09			
Lungs	4.78	1.46-15.60	0.009			
**Organ support**						
RRT	3.78	1.02-20.94	0.03			
Vasopressors	4.23	0.78-22.84	0.09			
Mechanical ventilation	10.66	1.22-93.12	0.03			
**Scores**						
MELD	1.09	1.01-1.18	0.04			
ACLF grade	11.69	2.92-46.85	0.0005			
CLIF-C ACLF	1.26	1.11-1.50	0.0006			
CLIF-C ACLF lactate	1.26	1.11-1.43	0.0004	1.26	1.11-1.43	0.0004
**Body composition parameters**						
L3SMI (cm^2/m^2)	3.41	0.23-48.97	0.40			
Sarcopenia (L3SMI cut-offs)	2.47	0.79-7.75	0.11			
SMRA (HU)	0.99	0.91-1.08	0.85			
IMATI (cm^2/m^2)	0.99	0.91-1.03	0.91			
VATI (cm^2/m^2)	1.00	0.98-1.03	0.54			
SATI (cm^2/m^2)	1.00	0.98-1.01	0.76			
VSR	1.56	0.62-4.68	0.45			
VAT-RA (HU)	1.03	0.98-1.10	0.18			
SAT-RA (HU)	1.04	1.01-1.08	0.05	1.01	0.98-1.07	0.18
SAT-RA (HU), according to (27)	1.71	0.46-6.39	0.42			

Table S5: Univariable and multivariable analyses of predictors of 28-days mortality in male patients from Lausanne cohort on day 3 (n=126)

	Univariable analysis			Multivariable analysis		
**Covariant**	OR	95% CI	p value	OR	95% CI	p value
**Characteristics**						
Age (years)	0.99	0.95-1.02	0.61			
Sex (male)	-	-	-			
BMI (kg/m^2)	1.01	0.93-1.09	0.77			
Ethnicity						
Caucasian	-	-	-			
Hispanic	1.98	0.67-3.98	0.19			
Other	0.87	0.19-7.12	0.49			
Clinical frailty score (score)	1.15	0.87-1.89)	0.12			
**Aetiology**						
Alcohol	-	-	-	-	-	-
Viral	2.76	1.12-6.76	0.03	2.51	0.79-7.97	0.11
Metabolic	0.85	0.21-3.44	0.82	2.50	0.55-11.28	0.23
Other	0.64	0.06-6.04	0.70	0.16	0.01-2.65	0.20
**Cause for ICU admission**						
Sepsis	-	-	-	-	-	-
Bleeding	0.43	0.18-1.03	0.06	0.48	0.15-1.56	0.22
Other	0.59	0.23-1.49	0.26	0.51	0.12-2.07	0.35
**Laboratory**						
Leukocytes (G/l)	1.00	0.96-1.04	0.90			
INR	8.71	1.20-63.10	0.03			
Bilirubin (mg/dl)	1.11	1.01-1.23	0.02			
AST (IU)	1.00	0.99-1.01	0.68			
Albumin (g/l)	1.06	0.98-1.16	0.12			
Creatinine (mg/dl)	1.45	1.04-2.02	0.02			
Sodium (mmol/l)	0.99	0.94-1.06	0.97			
Lactate (mmol/l)	1.55	1.20-2.01	0.0009			
Ammonia, (µmol/l)	1.00	0.99-1.01	0.85			
CRP (mg/l)	0.99	0.99-1.01	0.86			
**Organ failure**						
Liver	2.94	1.18-7.37	0.02			
Kidney	2.35	1.01-5.63	0.01			
Brain	1.68	0.87-6.64	0.12			
Coagulation	7.81	2.72-22.37	0.0002			
Circulation	4.20	1.88-9.41	0.0005			
Lungs	3.20	1.55-6.60	0.009			
**Organ support**						
RRT	4.82	1.63-14.21	0.005			
Vasopressors	4.20	1.88-9.41	0.0005			
Mechanical ventilation	2.36	1.15-5.23	0.02			
**Scores**						
MELD	1.13	1.07-1.20	<0.0001			
ACLF grade	3.10	1.90-5.02	<0.0001			
CLIF-C ACLF	1.17	1.10-1.25	<0.0001			
CLIF-C ACLF lactate	1.18	1.11-1.27	<0.0001	1.18	1.13-1.27	<0.0001
**Body composition parameters**						
L3SMI (cm^2/m^2)*	0.97	0.93-0.99	0.04	0.98	0.93-1.01	0.07
Sarcopenia (L3SMI cut-offs)*	4.11	1.64-10.31	0.002	1.95	0.95-6.79	0.06
SMRA (HU)	0.99	0.94-1.04	0.79			
IMATI (cm^2/m^2)	0.96	0.89-1.03	0.33			
VATI (cm^2/m^2)	0.98	0.97-1.01	0.13			
SATI (cm^2/m^2)	1.00	0.98-1.01	0.84			
VSR	0.75	0.36-1.52	0.42			
VAT-RA (HU) ¶	1.06	1.01-1.13	0.02	1.04	0.95-1.12	0.23
SAT-RA (HU) ¶	1.02	0.99-1.05	0.09	1.03	0.98-1.08	0.18
SAT-RA (HU), according to (27)	1.76	0.62-4.92	0.28			

*Not included in the same multivariable analysis to avoid collinearity

¶ Not included in the same multivariable analysis due to collinearity (Spearman: 0.79 DF 91, p<0.0001)

Table S6: Univariable and multivariable analyses of predictors of 28-day mortality in female patients from Lausanne cohort on day 3 (n=42)

	Univariable analysis			Multivariable analysis		
**Covariant**	OR	95% CI	p value	OR	95% CI	p value
**Characteristics**						
Age (years)	1.03	0.97-1.08	0.25			
Sex (male)	-	-	-			
BMI (kg/m^2)	0.97	0.85-1.12	0.75			
Ethnicity						
Caucasian	-	-	-			
Hispanic	1.91	0.21-10.41	0.23			
Other	0.98	0.18-7.93	0.71			
Clinical frailty scale (score)	1.11	0.81-2.23	0.17			
**Aetiology**						
Alcohol	-	-	-			
Viral	1.11	0.14-8.22	0.91			
Metabolic	1.11	0.19-6.290	0.89			
Other	1.67	0.24-11.26	0.59			
**Cause for ICU admission**						
Sepsis	-	-	-			
Bleeding	0.64	0.11-3.50	0.61			
Other	0.70	0.17-2.84	0.62			
**Laboratory**						
Leukocytes (G/l)	1.07	0.96-1.20	0.19			
INR	1.49	0.74-3.00	0.18			
Bilirubin (mg/dl)	1.07	1.01-1.20	0.04			
AST (IU)	1.00	0.99-1.01	0.16			
Albumin (g/l)	1.12	0.98-1.27	0.09			
Creatinine (mg/dl)	1.21	0.70-2.08	0.47			
Sodium (mmol/l)	1.00	0.91-1.11	0.91			
Lactate (mmol/l)	1.37	1.01-2.01	0.03			
Ammonia, (µmol/l)	1.00	0.99-1.01	0.29			
CRP (mg/l)	1.00	0.99-1.01	0.62			
**Organ failure**						
Liver	6.89	1.18-40.27	0.03			
Kidney	2.50	0.85-11.21	0.08			
Brain	1.26	0.85-4.21	0.20			
Coagulation	11.36	1.18-109.02	0.02			
Circulation	1.94	1.02-6.94	0.03			
Lungs	2.22	1.13-4.38	0.03			
**Organ support**						
RRT	5.73	1.21-61.12	0.02			
Vasopressors	1.94	1.02-6.94	0.03			
Mechanical ventilation	12.85	1.47-112.17	0.02			
**Scores**						
MELD	1.10	1.02-1.19	0.01			
ACLF grade	2.42	1.15-5.12	0.009			
CLIF-C ACLF	1.21	1.06-1.37	0.002			
CLIF-C ACLF lactate	1.21	1.06-1.37	0.002	1.17	1.05-1.32	0.004
**Body composition parameters**						
L3SMI (cm^2/m^2)*	2.60	0.16-45.11	0.50			
Sarcopenia (L3SMI cut-offs)	2.56	0.69-9.49	0.13			
SMRA (HU)	1.03	0.93-1.14	0.52			
IMATI (cm^2/m^2)	0.94	0.85-1.05	0.33			
VATI (cm^2/m^2)	1.00	0.97-1.03	0.68			
SATI (cm^2/m^2)	0.99	0.98-1.01	0.65			
VSR	1.49	0.49-4.46	0.47			
VAT-RA (HU)	1.05	0.96-1.14	0.24			
SAT-RA (HU)	1.04	0.99-1.09	0.09	1.03	0.94-1.14	0.40
SAT-RA (HU), according to (27)	2.50	0.66-11.01	0.15			

Table S7: Main characteristics comparison on day 1 between patients included in the overall Lausanne cohort (n=192) and patients included in the Villejuif external cohort (n=58). Continuous and categorical variables are expressed respectively in median (interquartile range) and N (percentages). Comparisons were performed using the Student's t-test or Mann-Whitney U test for quantitative variables or Chi-Square and Fisher exact tests for categorical variables as appropriate

	Overall population included in the final analyses (n=192)	External cohort from Villejuif (N=58)	p
**Characteristics**			
Age (years)	62.0 (53.2-70.0)	55.3 (48.7-62.9)	0.0002
Sex (male)	141 (73.5)	46 (79.3)	0.36
BMI (kg/m²)	25.8 (22.3-31.2)	28.0 (24.0-32.0)	0.09
Ethnicity<br>Causasian<br>Hispanic<br>Other	 161 (83.8)<br>16 (8.3)<br>15 (7.8)	 38 (65.5)<br>11 (19.0)<br>9 (15.5)	<0.0001
Clinical frailty scale (score)	4.0 (3.0-4.0)	3.0 (3.0-4.0)	0.21
**Aetiology**			
Alcohol<br>Viral<br>Metabolic<br>Other	129 (67.2)<br>34 (17.7)<br>18 (9.4)<br>11 (5.7)	46 (79.3)<br>3 (5.2)<br>7 (12.1)<br>2 (3.5)	0.02
**Precipitating event**			
Sepsis<br>Bleeding<br>Other	79 (41.1)<br>63 (32.8)<br>50 (26.1)	23 (39.7)<br>16 (27.6)<br>19 (32.7)	0.23
**Laboratory on day 1**			
Leukocytes (G/l)	15.0 (10.1-20.8)	11.5 (6.4-14.8)	0.001
INR	1.5 (1.3-1.8)	2.5 (2.1-3.6)	<0.0001
Bilirubin (mg/dl)	5.3 (3.7-8.2)	12.8 (4.2-19.6)	<0.0001
AST (IU)	93.0 (48.0-299.5)	95.0 (65.0-197.5)	0.11
Albumin (g/l)	27.0 (23.2-31.0)	28.0 (24.0-32.0)	0.26
Creatinine (mg/dl)	1.4 (1.0-2.1)	1.5 (1.2-2.3)	0.42
Sodium (mmol/l)	138.0 (134.0-141.0)	138.0 (134.0-142.0)	0.93
Lactate (mmol/l)	4.1 (2.4-7.4)	2.7 (1.5-4.3)	0.20
CRP (mg/l)	55.0 (20.0-112.5)	35.0 (18.0-72)	0.02
**ACLF grade on day 1**			
0<br>1<br>2<br>3	0 (0)<br>26 (13.6)<br>59 (30.7)<br>107 (55.7)	0 (0)<br>7 (12.1)<br>15 (25.9)<br>36 (62.0)	<0.0001
**Scores on day 1**			
MELD	21.9 (15.1-27.9)	31.0 (25.0-37.0)	<0.0001
CLIF-C ACLF	67.5 (51.8-72.7)	77.5 (73.5-82.9)	<0.0001
CLIF-C ACLF-lactate	71.5 (64.1-80.2)	78.9 (72.2-87.8)	<0.0001
**Outcome**			
28-day survival, % (95%CI)	58.2 (51.2-65.2)	41.4 (28.7-54.0)	<0.0001
**Body composition parameters**			
L3SMI (cm²/m²)	43.2 (37.1-50.1)	41.6 (36.2-49.4)	0.71
Sarcopenia according to L3SMI sex specific cut-offs	121 (63.0)	41 (70.7)	0.25
SMRA (HU)	36.0 (31.0-41.0)	30.3 (25.1-37.1)	0.0004
IMATI (cm²/m²)	6.3 (3.9-10.0)	4.0 (3.2-5.2)	<0.0001
VATI (cm²/m²)	44.3 (24.4-69.9)	30.4 (14.2-48.7)	<0.0001
SATI (cm²/m²)	48.6 (27.0-70.3)	43.6 (29.7-74.6)	0.48
VSR	0.9 (0.6-1.4)	0.7 (0.4-1.1)	0.009
VAT-RA (HU)	-81.2 (-88.5 to -75.8)	-72.6 (-79.1 to -69.4)	<0.0001
SAT-RA (HU)	-86.6 (-95.7 to -76.8)	-77.6 (-86.1 to -65.5)	<0.0001
